# Supplementary material for: Synthesis of (1,10-Phenanthroline-κ2N,N′)(β2-Methyl- and β2-PhenylAlaninate-κ2N,O)Copper(II) Nitrate Complexes and Their Antiproliferative Activity on MCF-7 and A549 Cancer Cell Lines
Source: Molecules. 2025 Jan 31;30(3):634. doi: 10.3390/molecules30030634 (PMC11820680; doi:10.3390/molecules30030634)

Supplementary information for

Synthesis of (1,10-Phenanthroline-κ^2N,N')(β^2-Methyl- and β^2-PhenylAlaninate-κ^2N,O)Copper(II) Nitrate Complexes and their Antiproliferative Activity on MCF-7 and A549 Cancer Cell Lines.

Leticia Chavelas-Hernández[1], Luis G. Hernández-Vázquez[1,*], Jonathan R. Valdez-Camacho[1,2], Adrián Espinoza-Guillén[3], Carlos A. Tavira-Montalván[4], Angélica Meneses-Acosta[4], Eusebio Juaristi[5,6], Lena Ruiz-Azuara[3], Jaime Escalante[1,*]

[1] Centro de Investigaciones Químicas-IICBA, Universidad Autónoma del Estado de Morelos. Av. Universidad 1001, Cuernavaca 62209 Morelos México; letychh@gmail.com (L.Ch.-H); valca@uaem.mx (J.R.V.-C.). [2] Departamento de Química, Universidad Autónoma Metropolitana-Iztapalapa, San Rafael Atlixco 186, Col. Vicentina, Iztapalapa, C.P. 09340, Ciudad de México, México; [3] Facultad de Química, Departamento de Química Inorgánica y Nuclear, Universidad Nacional Autónoma de México, Av. Universidad 3000, México DF 04510, México; adrianeg@quimica.unam.mx (A-E.-G.); lenar701@gmail.com (L.R.-A.). [4] Facultad de Farmacia, Universidad Autónoma del Estado de Morelos, Av. Universidad 1001, Cuernavaca, 62209 Morelos México; carlos.taviramon@uaem.edu.mx (C.A.T.-M.). [5] Departamento de Química, Centro de Investigación y de Estudios Avanzados, Avenida I.P.N. 2508, Ciudad de México 07360, México; juaristi@relaq.mx (E.J.). [6] El Colegio Nacional, Luis González Obregón 23, Centro Histórico, 06020 Ciudad de México, México.
* Correspondence: luishdezv@uaem.mx (L.G.H.-V.); jaime@uaem.mx (J.E.);
Tel.: +52-77-7329-7997 (J.E.) (L.G.H.-V.)

Figure S1. ^{1}H NMR (200 MHz CD_3OD) for *(rac)*-3-Amino-2-methylpropanoic acid chlorohydrate **1**.

Figure S2. ^{13}C NMR (50 MHz CD_3OD) for *(rac)*-3-Amino-2-methylpropanoic acid chlorohydrate **1**.

Figure S3. ^{1}H NMR (500 MHz CD_3OD) for *(rac)*-3-Amino-2-benzylpropanoic acid chlorohydrate **2**.

Figure S4. ^{13}C NMR (125 MHz CD_3OD) for *(rac)*-3-Amino-2-benzylpropanoic acid chlorohydrate **2**.

Figure S5. ATR FT-IR spectra for background.

Figure S6. ATR FT-IR spectra for $[Cu(H_2O)(phen)L1]NO_3{\cdot}2H_2O$ (**3**).

Figure S7. ATR FT-IR spectra $[Cu(EtOH)(phen)L2]NO_3$ (**4**).

Figure S8. UV-Vis spectra for background in methanol.

Figure S9. UV-Vis spectra for background in water.

Figure S10. UV-Vis spectra for $[Cu(H_2O)(phen)L1]NO_3{\cdot}2H_2O$ (**3**) in methanol.

Figure S11. UV-Vis spectra for $[Cu(H_2O)(phen)L1]NO_3{\cdot}2H_2O$ (**3**) in water.

Figure S12. UV-Vis spectra for $[Cu(H_2O)(phen)L1]NO_3{\cdot}2H_2O$ (**3**) in DMEM $1x10^{-3}$ M.

Figure S13. UV-Vis spectra for $[Cu(H_2O)(phen)L1]NO_3{\cdot}2H_2O$ (**3**) in PBS $1x10^{-3}$ M.

Figure S14. UV-Vis spectra for $[Cu(EtOH)(phen)L2]NO_3$ (**4**) in methanol.

Figure S15. UV-Vis spectra for $[Cu(EtOH)(phen)L2]NO_3$ (**4**) in water.

Figure S16. UV-Vis spectra for $[Cu(EtOH)(phen)L2]NO_3$ (**4**) in in DMEM $1X10^{-3}$ M.

Figure S17. UV-Vis spectra for $[Cu(EtOH)(phen)L2]NO_3$ (**4**) in PBS $1X10^{-3}$ M.

Figure S18. UV-Vis spectra for $[Cu(H_2O)(phen)L1]NO_3{\cdot}2H_2O$ (**3**) in water $3.75x10^{-3}$.

Figure S19. UV-Vis spectra for $[Cu(EtOH)(phen)L2]NO_3$ (**4**) in water $3.75x10^{-3}$.

Figure S20. UV-Vis spectra for copper(II) nitrate in PBS, DMEM, MeOH and water $1X10^{-3}$ M.

Figure S21. UV-Vis spectra for copper(II) nitrate + 1,10-phenanthroline in PBS, DMEM, MeOH and water $1X10^{-3}$ M.

Figure S22. UV-Vis spectra for copper(II) nitrate + L1 in PBS, DMEM, MeOH and water $1X10^{-3}$ M.

Figure S23. UV-Vis spectra for copper(II) nitrate + L2 in PBS, DMEM, MeOH and water $1X10^{-3}$ M.

Figure S24. Half-wave potential for $[Cu(H_2O)(phen)L1]NO_3{\cdot}2H_2O$ (**3**).

Figure S25. Half-wave potential for $[Cu(EtOH)(phen)L2]NO_3$ (**4**).

Figure S26. Half-maximal inhibitory concentration for $[Cu(H_2O)(phen)L1]NO_3{\cdot}2H_2O$ (**3**).

Figure S27. Half-maximal inhibitory concentration for $[Cu(EtOH)(phen)L2]NO_3$ (**4**).

Figure S28. HR MS(ESI^+) $[Cu(H_2O)(phen)L1]NO_3{\cdot}2H_2O$ (**3**).

Figure S29. HR MS(ESI^+) $[Cu(EtOH)(phen)L2]NO_3$ (**4**).

Table S1. Crystal data for $[Cu(H_2O)(phen)L1]NO_3{\cdot}2H_2O$ (**3**).

Table S2. Bond Lengths for $[Cu(H_2O)(phen)L1]NO_3{\cdot}2H_2O$ (**3**).

Table S3. Bond Angles for $[Cu(H_2O)(phen)L1]NO_3{\cdot}2H_2O$ (**3**).

Table S4. Torsion Angles for $[Cu(H_2O)(phen)L1]NO_3{\cdot}2H_2O$ (**3**)

Table S5. Crystal data and structure refinement for $[Cu(EtOH)(phen)L2]NO_3$ (**4**).

Table S6. Bond Lengths for $[Cu(EtOH)(phen)L2]NO_3$ (**4**).

Table S7. Bond Angles for $[Cu(EtOH)(phen)L2]NO_3$ (**4**).

Table S8. Torsion Angles for $[Cu(EtOH)(phen)L2]NO_3$ (**4**).

CheckCIF for $[Cu(H_2O)(phen)L1]NO_3{\cdot}2H_2O$ (**3**).

CheckCIF for $[Cu(EtOH)(phen)L2]NO_3$ (**4**).

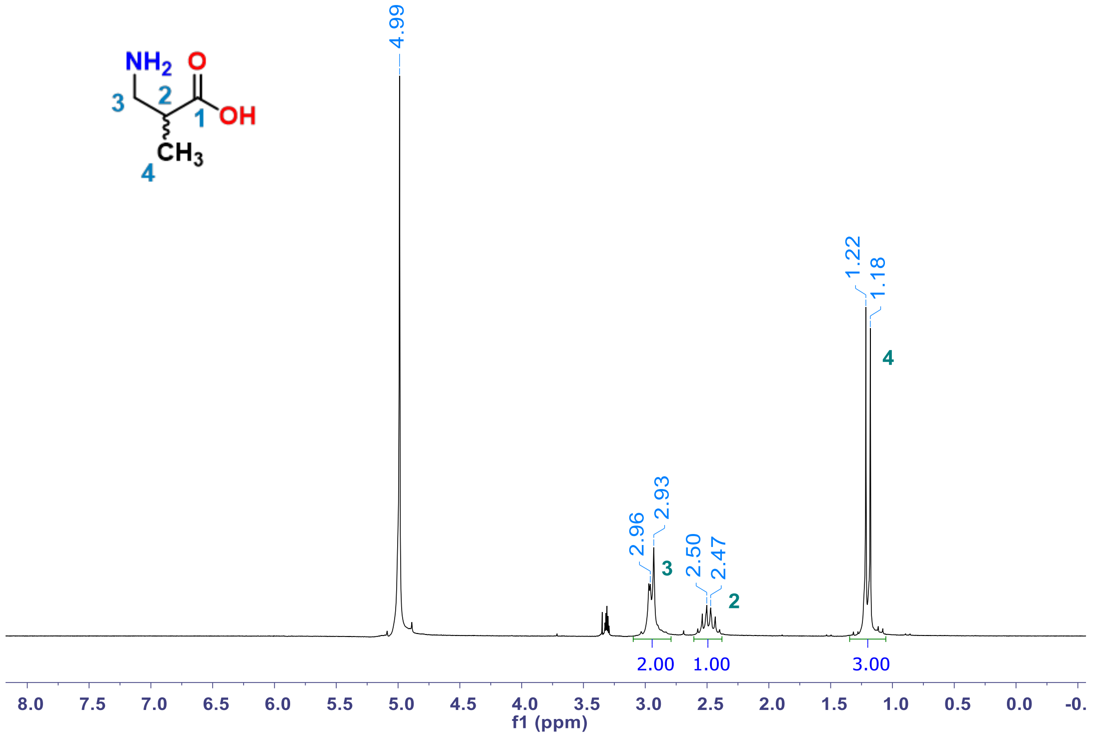

Figure S1. ^{1}H NMR (200 MHz CD_3OD) for *(rac)*-3-Amino-2-methylpropanoic acid chlorohydrate **1**.

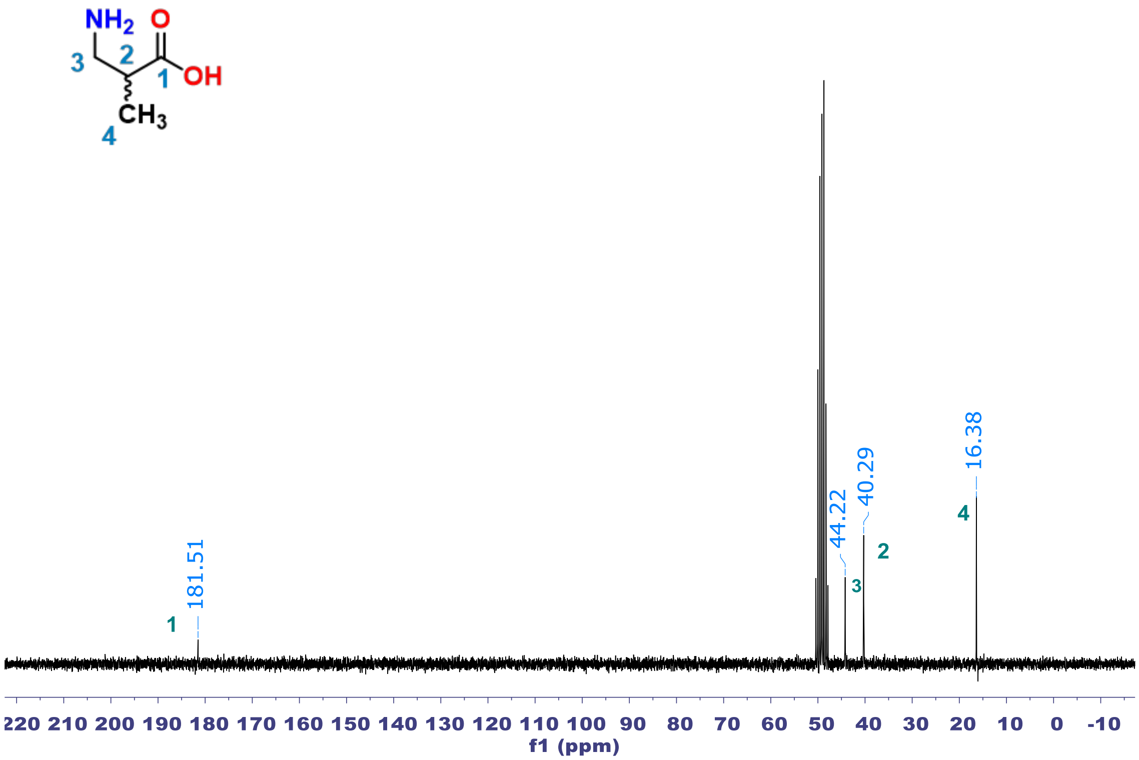

Figure S2. ^{13}C NMR (50 MHz CD_3OD) for *(rac)*-3-Amino-2-methylpropanoic acid chlorohydrate **1**.

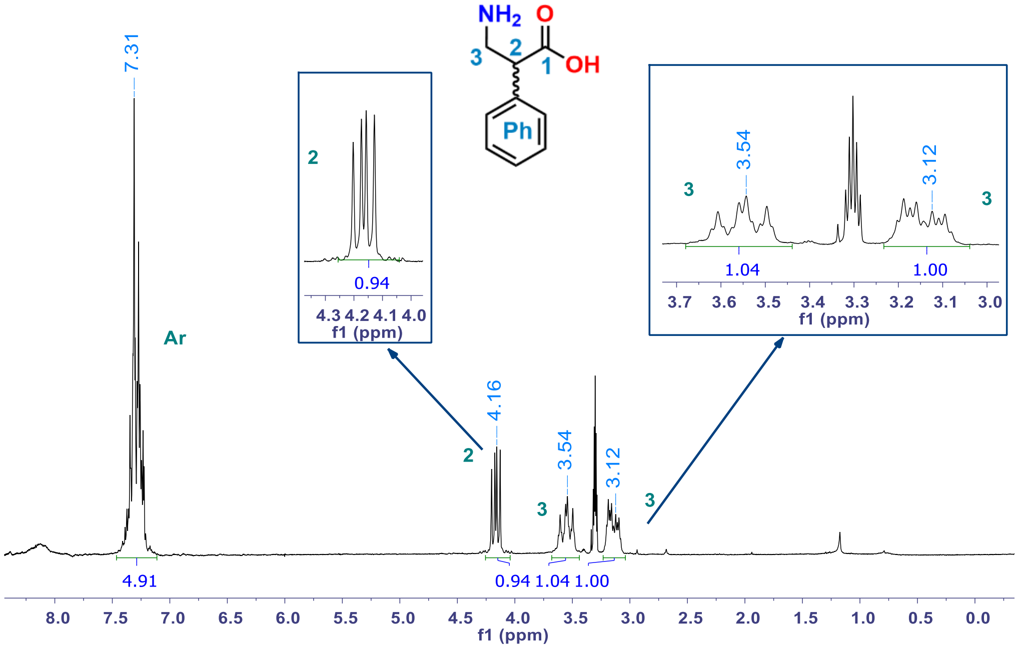

Figure S3. ^{1}H NMR (500 MHz CD_3OD) for *(rac)*-3-Amino-2-benzylpropanoic acid chlorohydrate **2**.

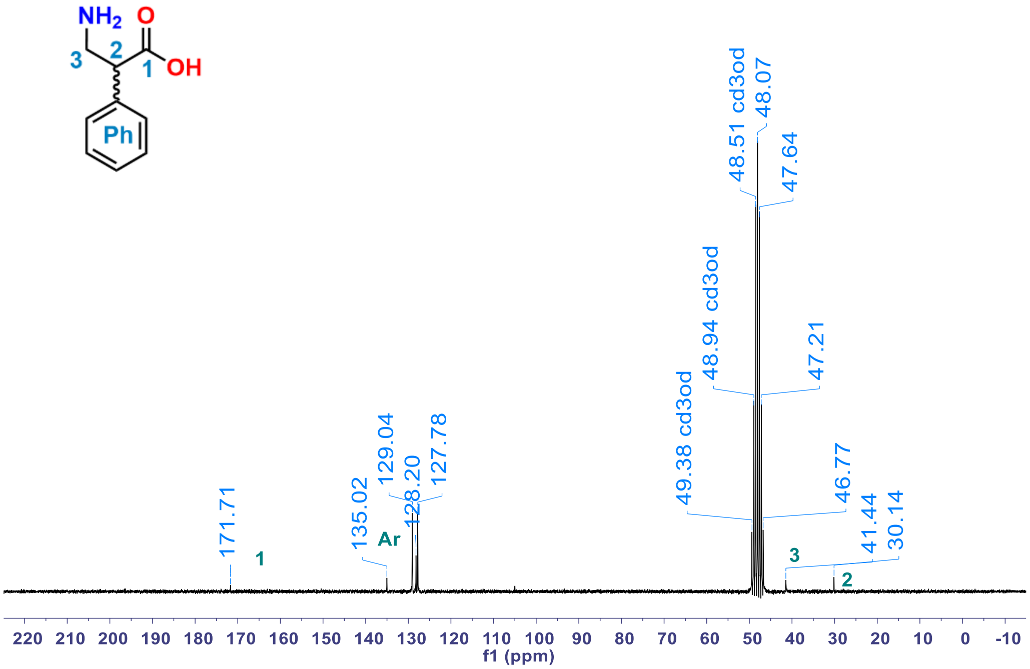

Figure S4. ^{13}C NMR (125 MHz CD_3OD) for *(rac)*-3-Amino-2-benzylpropanoic acid chlorohydrate **2**.

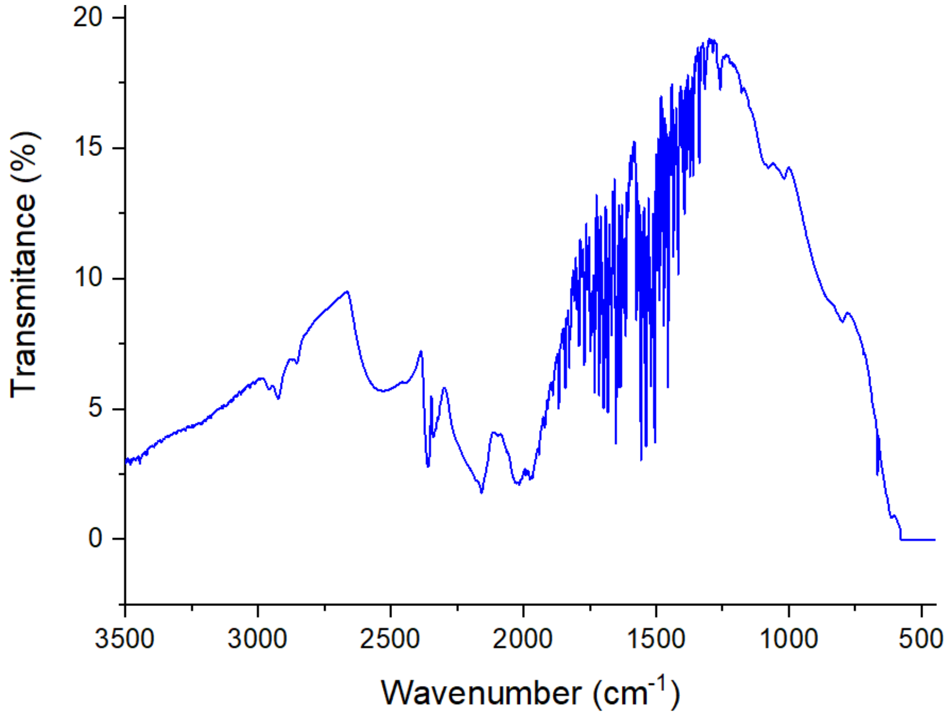

Figure S5. FT-IR spectra for background.

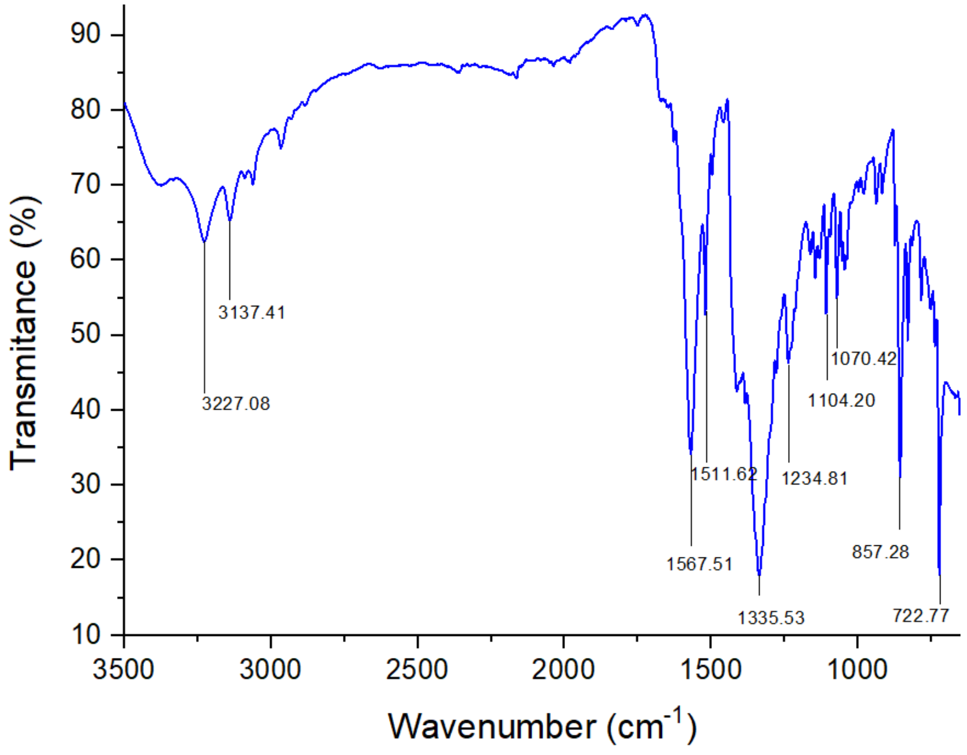

Figure S6. ATR FT-IR spectra for $[Cu(H_2O)(phen)L1]NO_3·2H_2O$ (**3**).

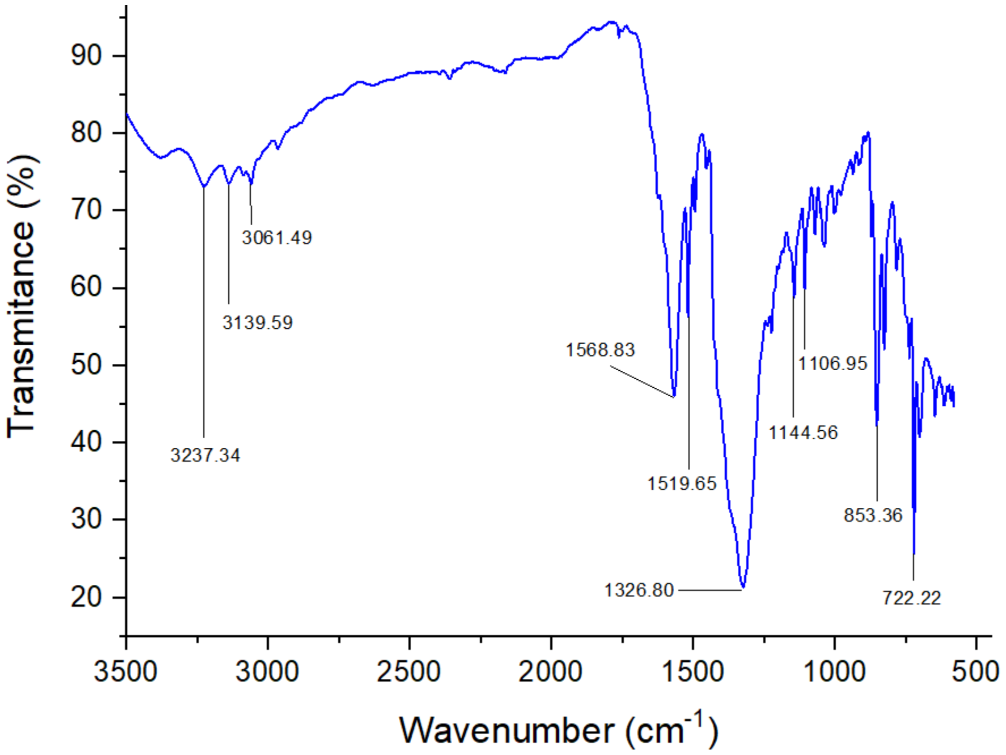

Figure S7. ATR FT-IR spectra for [Cu(EtOH)(phen)L2]NO_3 (**4**).

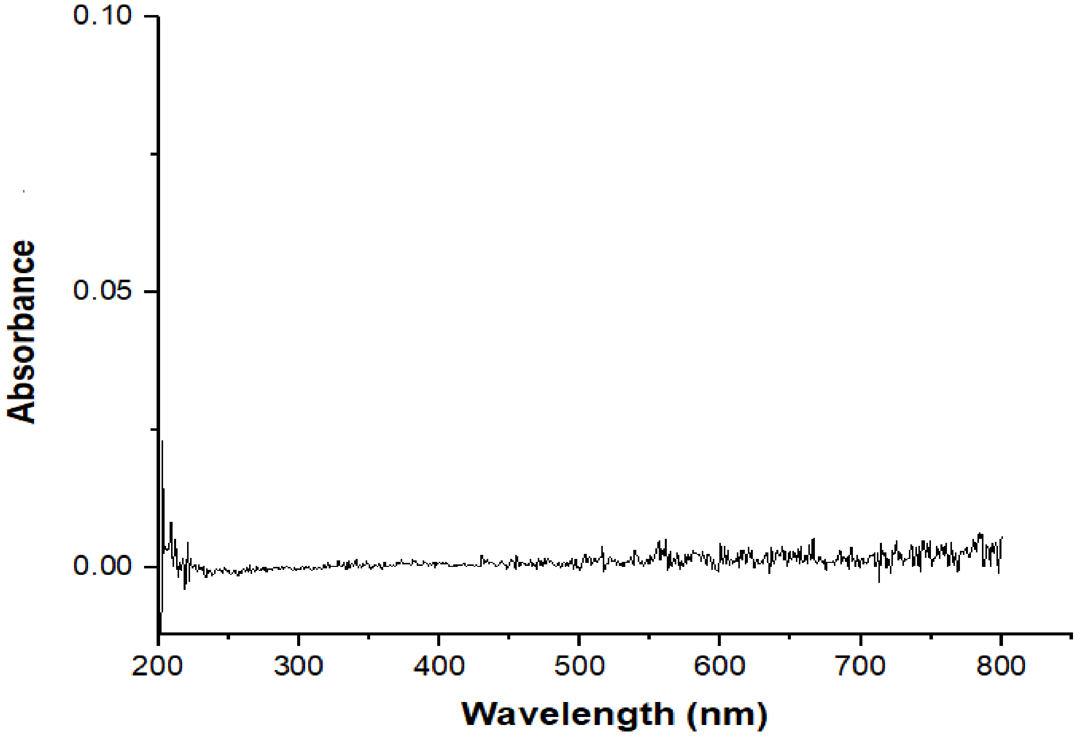

Figure S8. UV-Vis spectra for background in methanol.

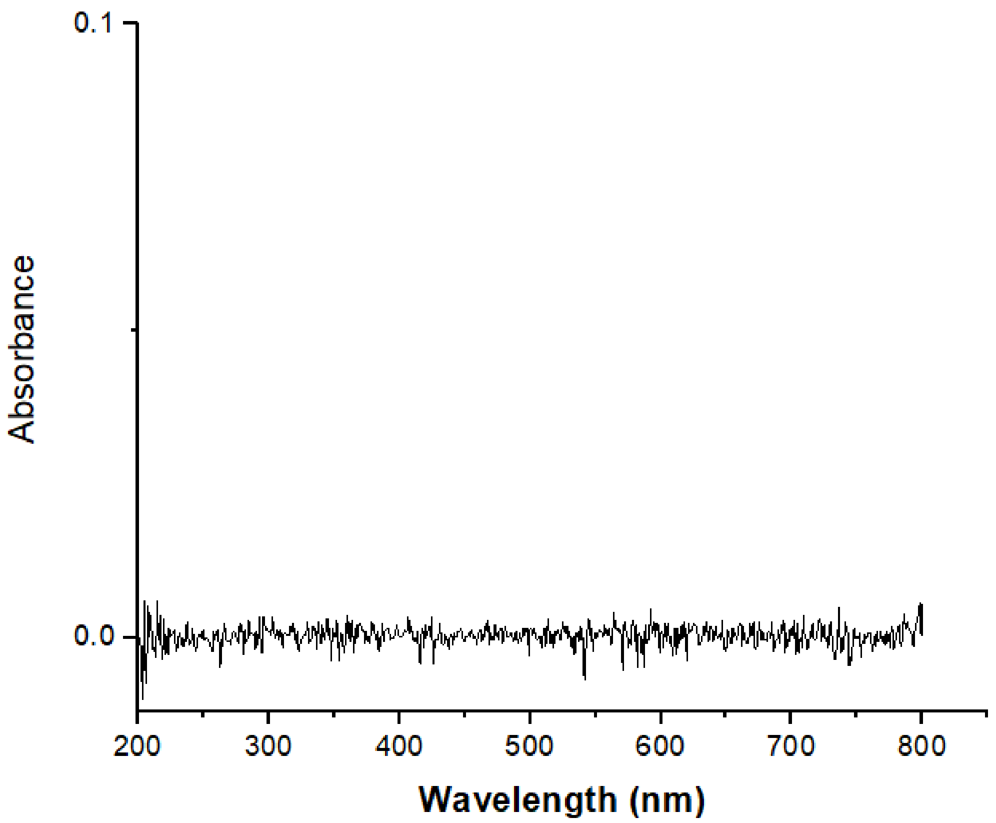

Figure S9. UV-Vis spectra for background in water.

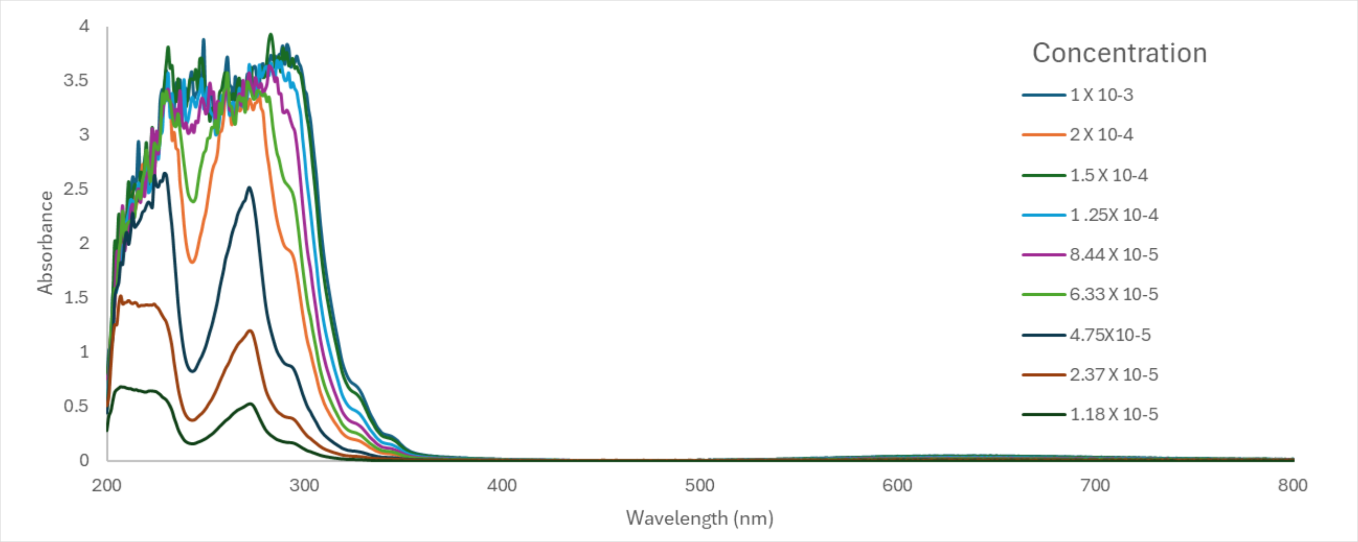

Figure S10. UV-Vis spectra for $[Cu(H_2O)(phen)L1]NO_3{\cdot}2H_2O$ (**3**) in methanol.

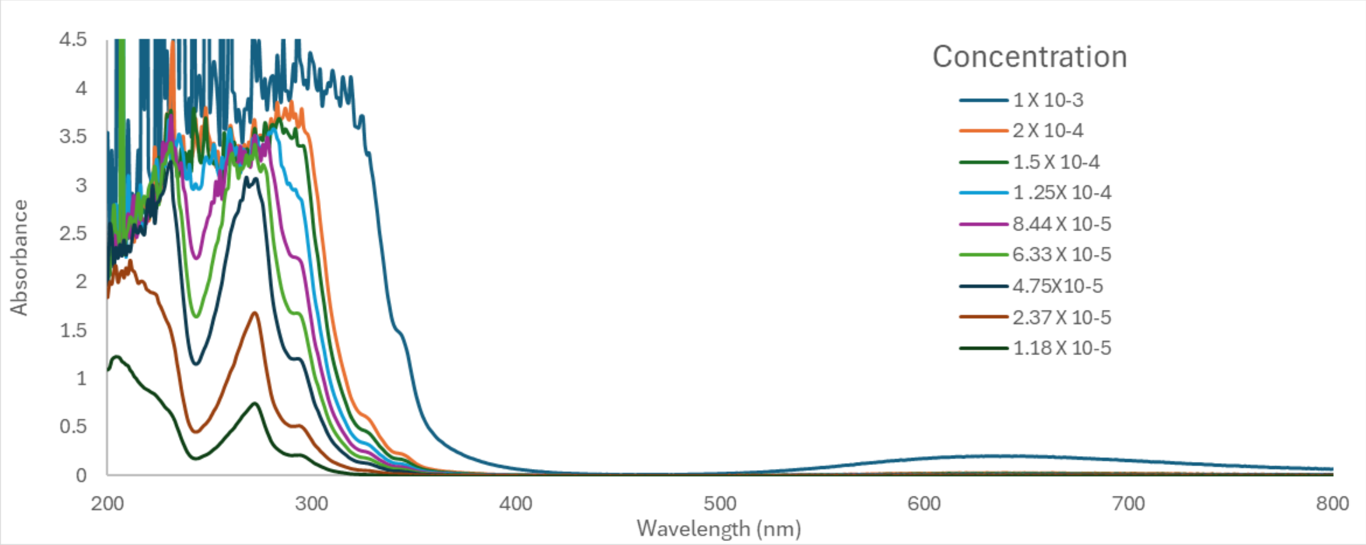

Figure S11. UV-Vis spectra for [Cu(H_2O)(phen)L1]NO_3·2H_2O (**3**) in water.

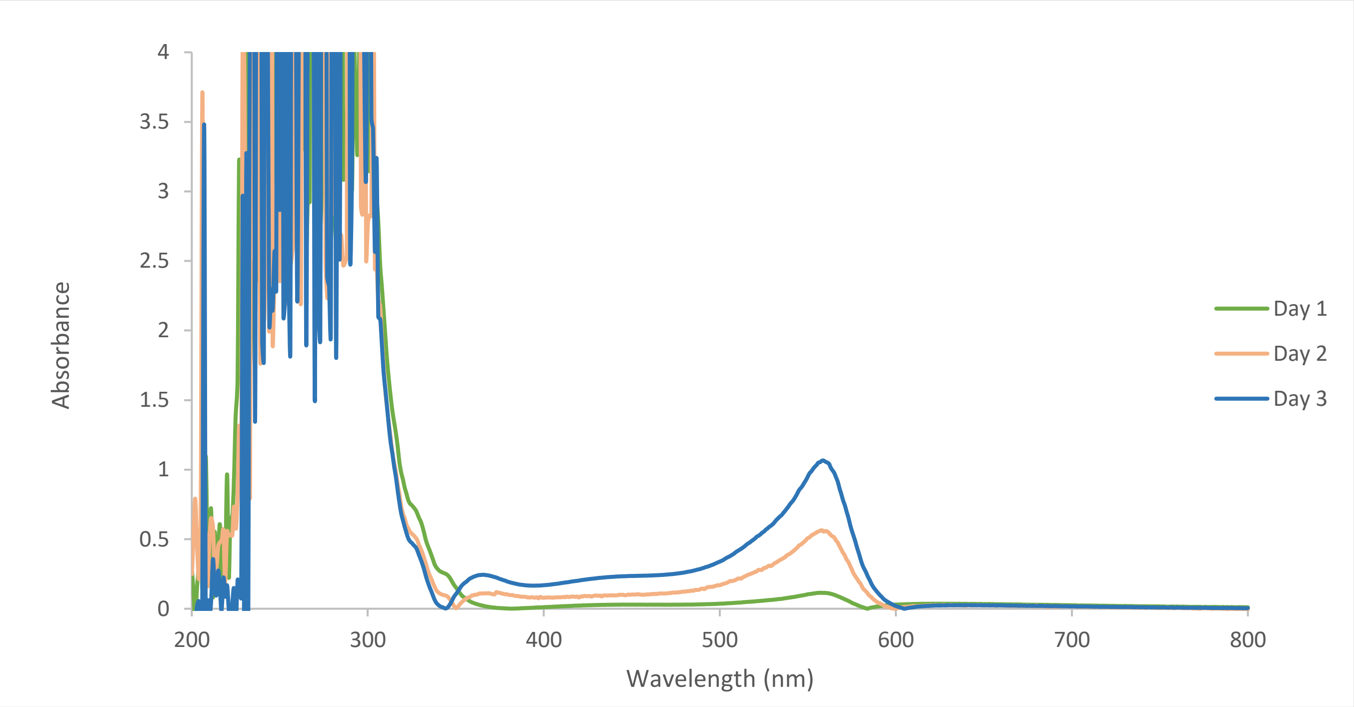

Figure S12. UV-Vis spectra for [Cu(H_2O)(phen)L1]NO_3·2H_2O (**3**) in DMEM 1x10^{-3} M.

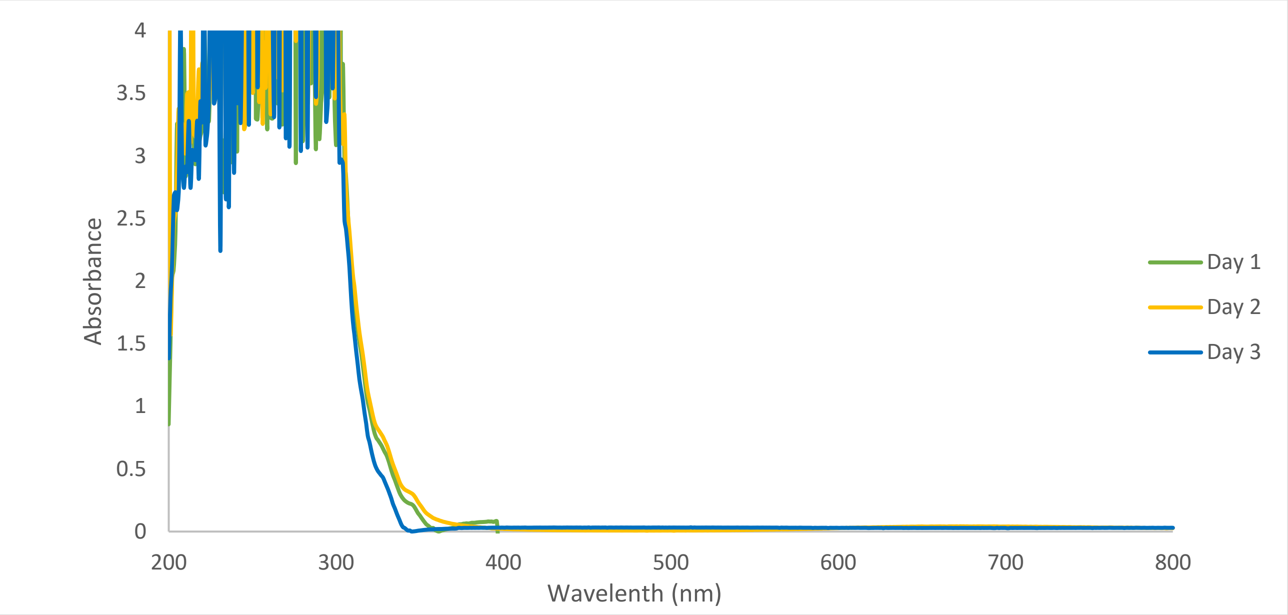

Figure S13. UV-Vis spectra for $[Cu(H_2O)(phen)L1]NO_3{\cdot}2H_2O$ (**3**) in PBS $1x10^{-3}$ M.

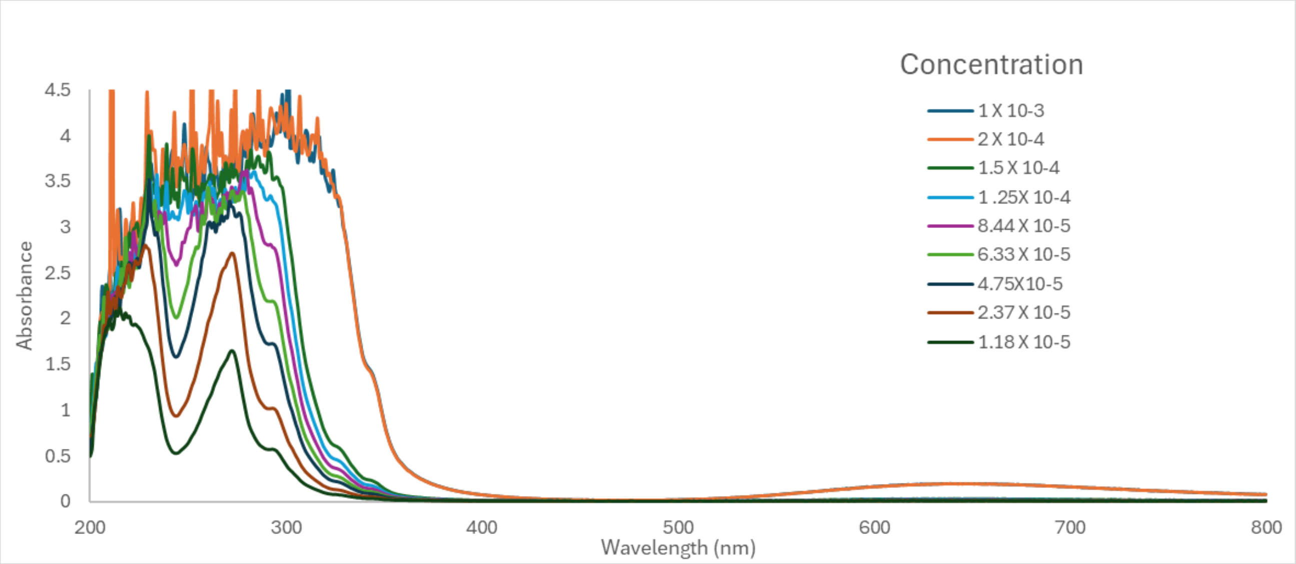

Figure S14. UV-Vis spectra for $[Cu(EtOH)(phen)L2]NO_3$ (**4**) in methanol.

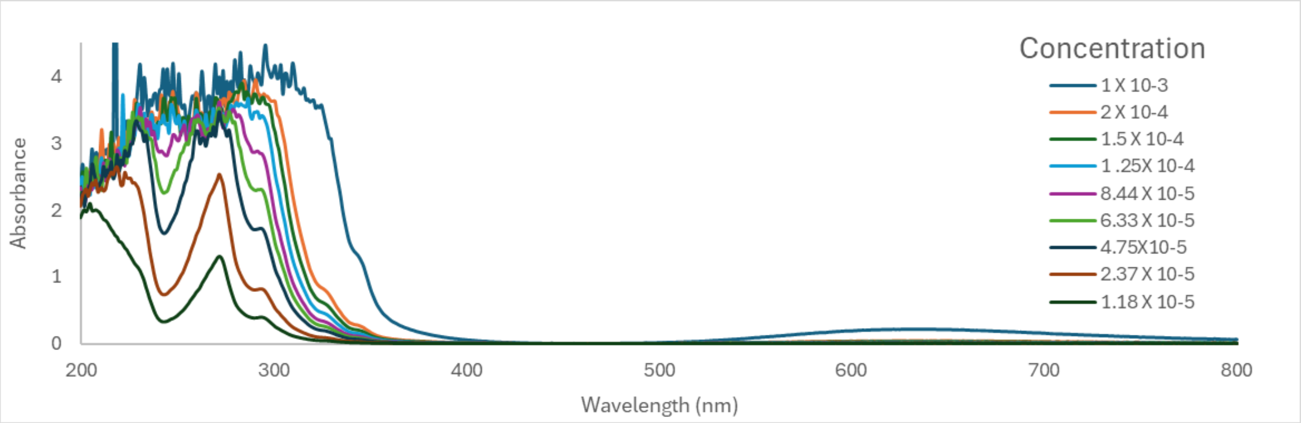

Figure S15. UV-Vis spectra for [Cu(EtOH)(phen)L2]NO_3 (**4**) in water.

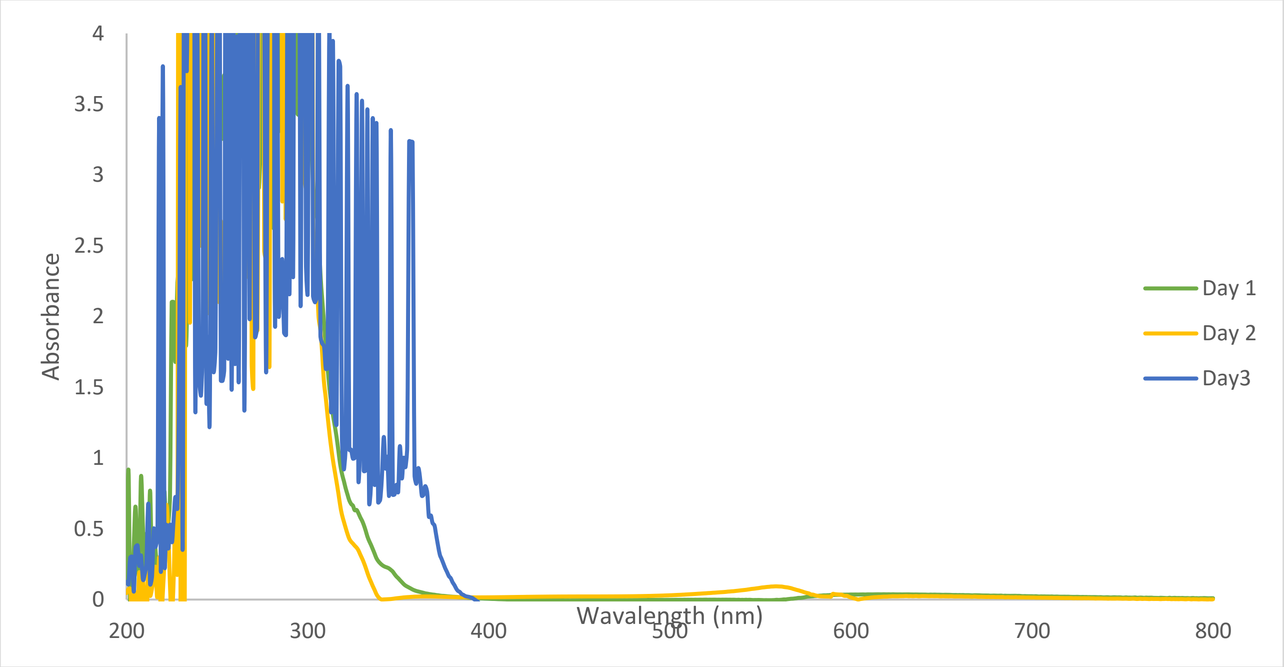

Figure S16. UV-Vis spectra for [Cu(EtOH)(phen)L2]NO_3 (**4**) in in DMEM 1X10^{-3} M.

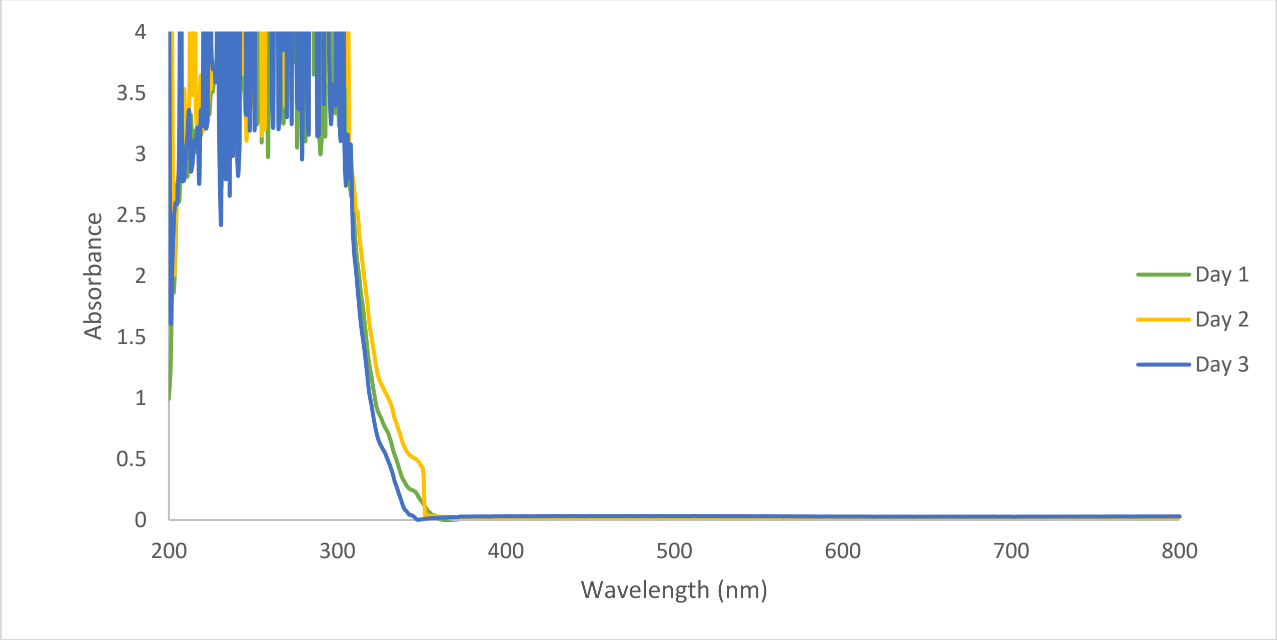

Figure S17. UV-Vis spectra for [Cu(EtOH)(phen)L2]NO_3 (**4**) in PBS 1X10^{-3} M.

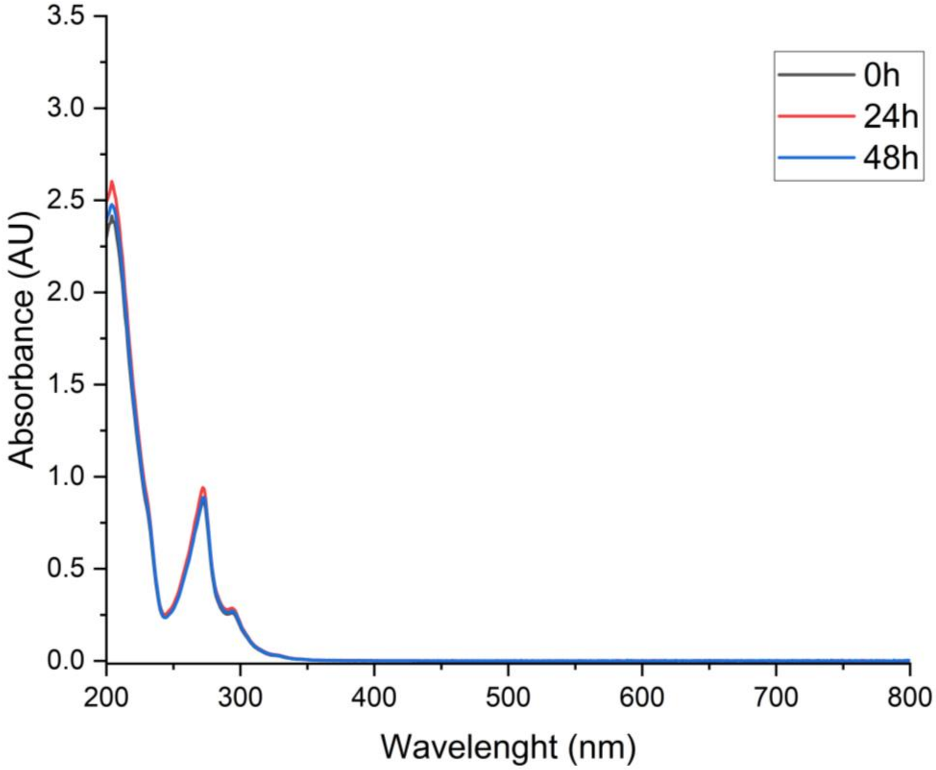

Figure S18. UV-Vis spectra for [Cu(H_2O)(phen)L1]NO_3·$2H_2O$ (**3**) in water 3.75x10^{-3}.

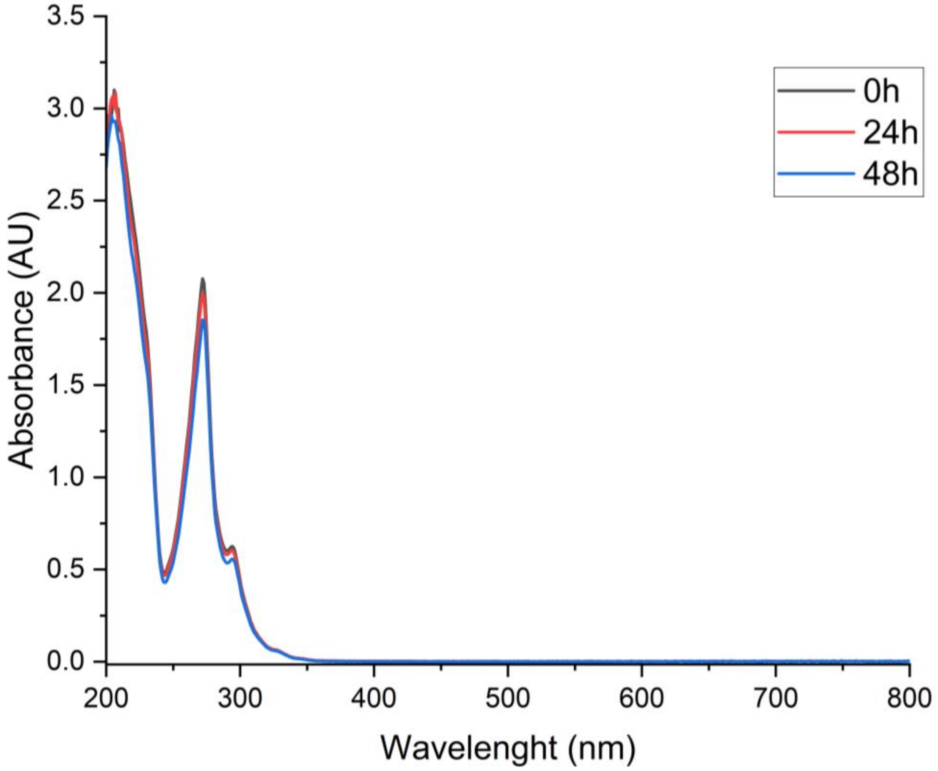

Figure S19. UV-Vis spectra for [Cu(EtOH)(phen)L2]NO_3 (**4**) in water 3.75x10^{-3}.

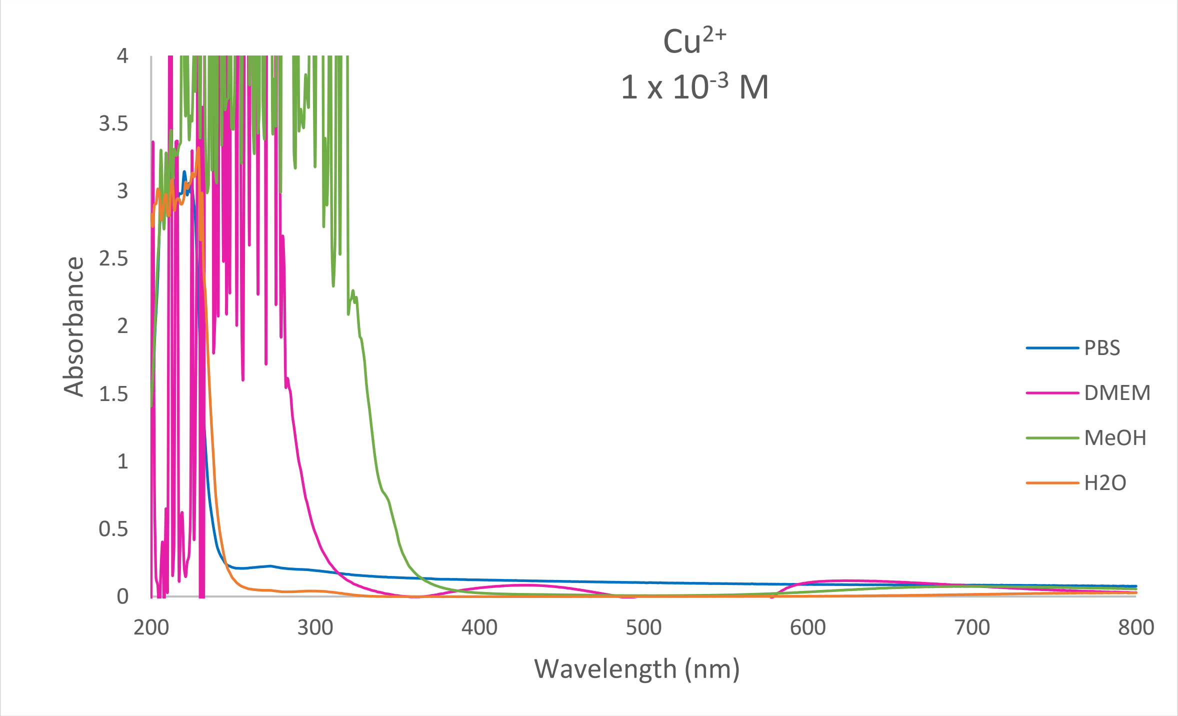

Figure S20. UV-Vis spectra for copper(II) nitrate in PBS, DMEM, MeOH and water 1X10^{-3} M.

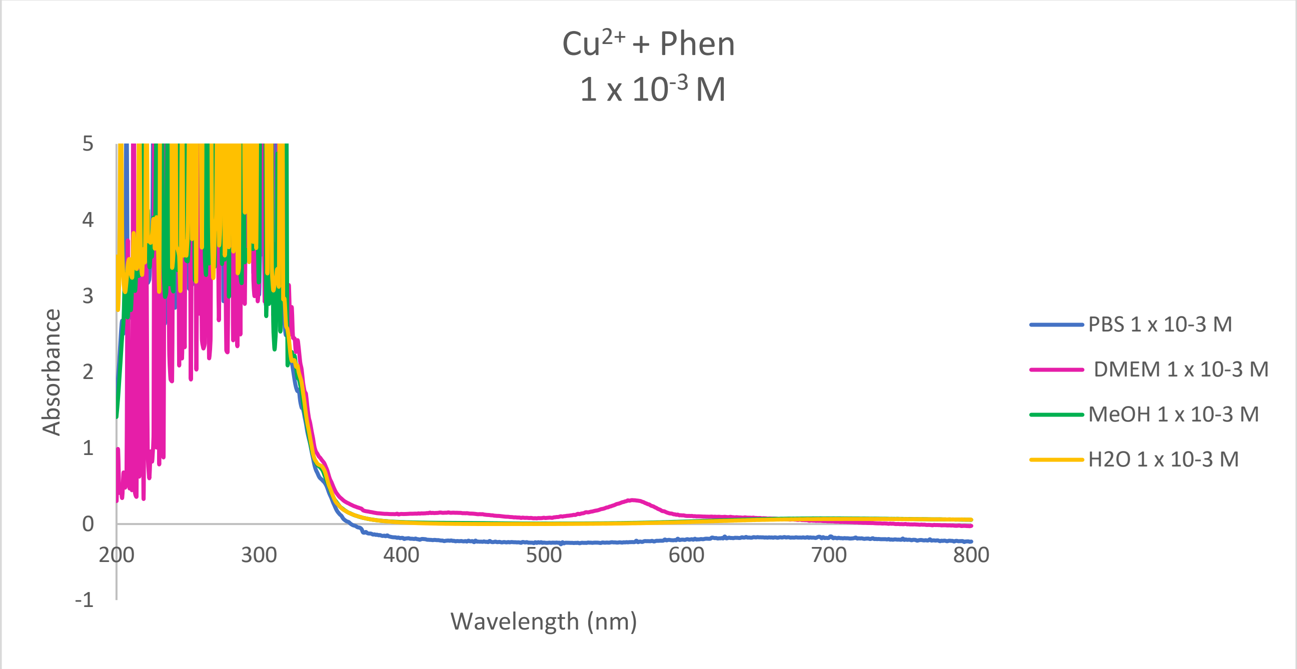

Figure S21. UV-Vis spectra for copper(II) nitrate + 1,10-phenanthroline in PBS, DMEM, MeOH and water 1X10^{-3} M.

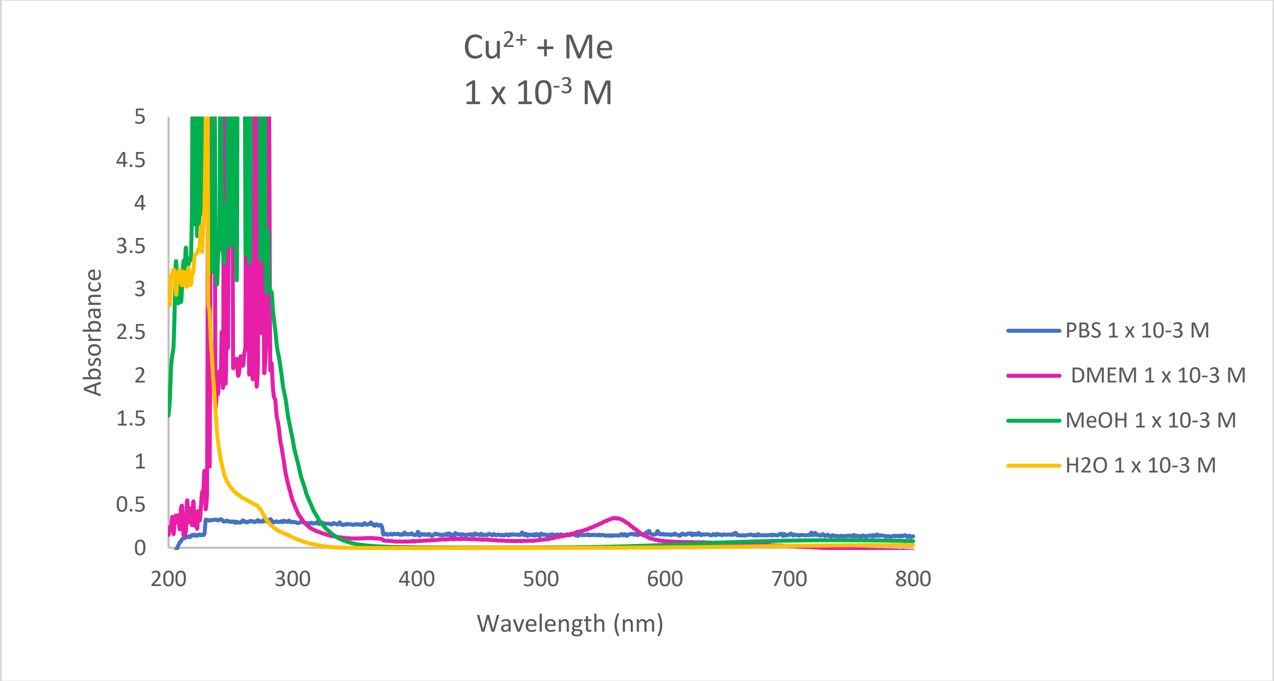

Figure S22. UV-Vis spectra for copper(II) nitrate + L1 in PBS, DMEM, MeOH and water 1X10^{-3} M.

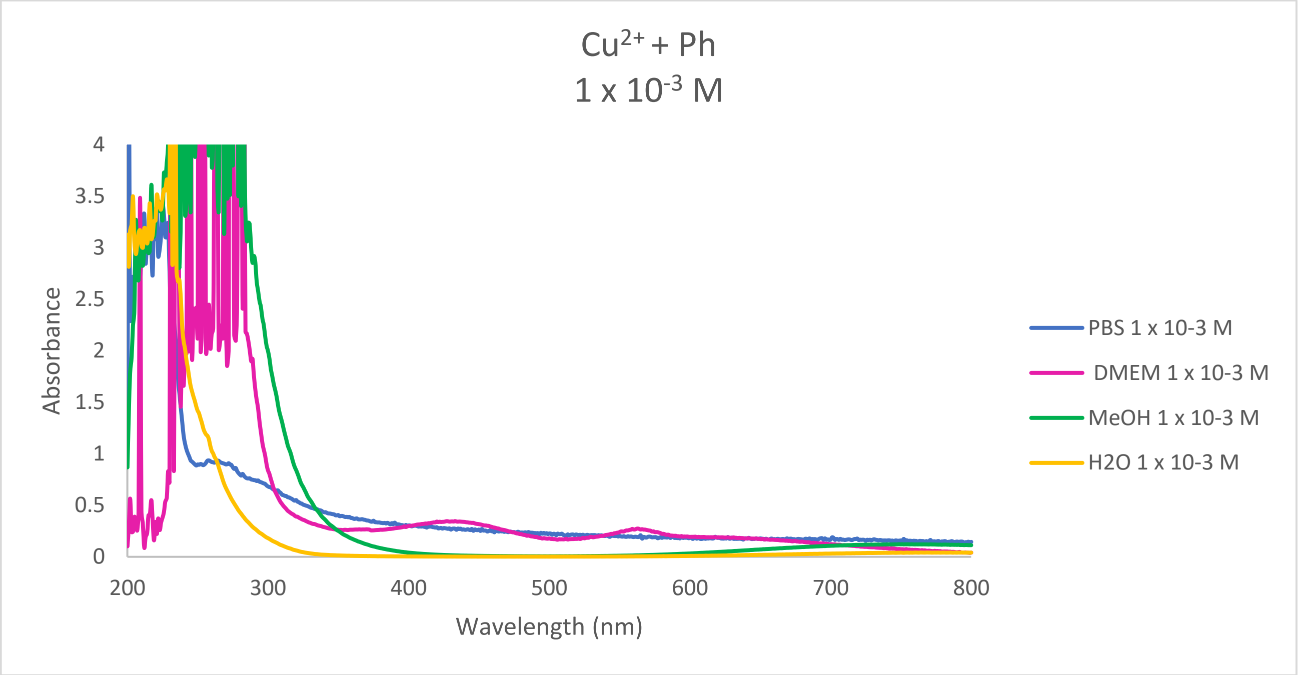

Figure S23. UV-Vis spectra for copper(II) nitrate + L2 in PBS, DMEM, MeOH and water 1X10^{-3} M.

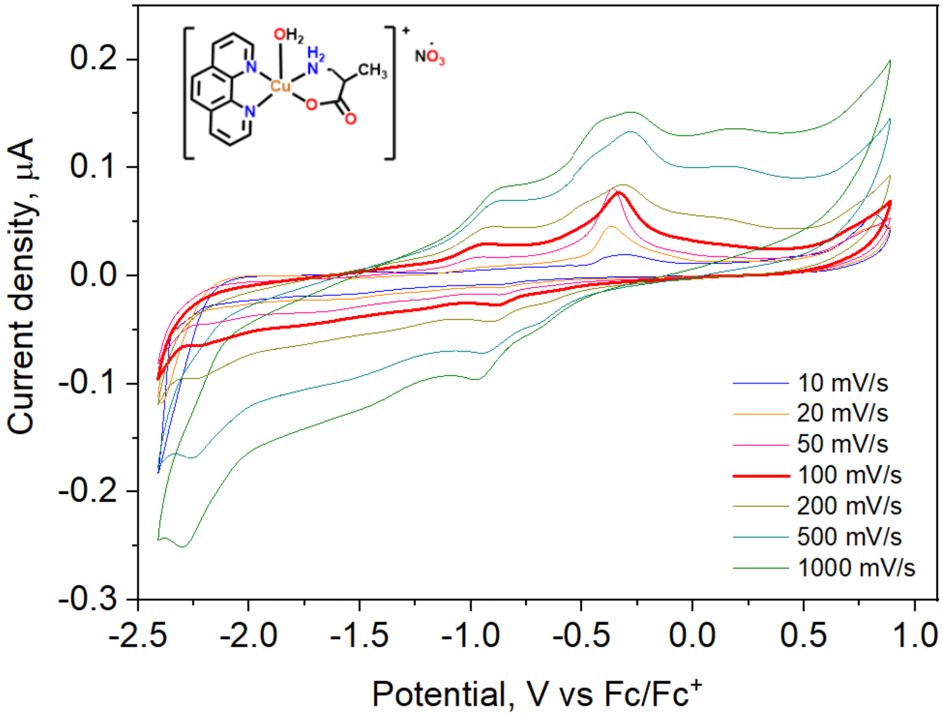

Figure S24. Half-wave potential for $[Cu(H_2O)(phen)L1]NO_3{\cdot}2H_2O$ (**3**).

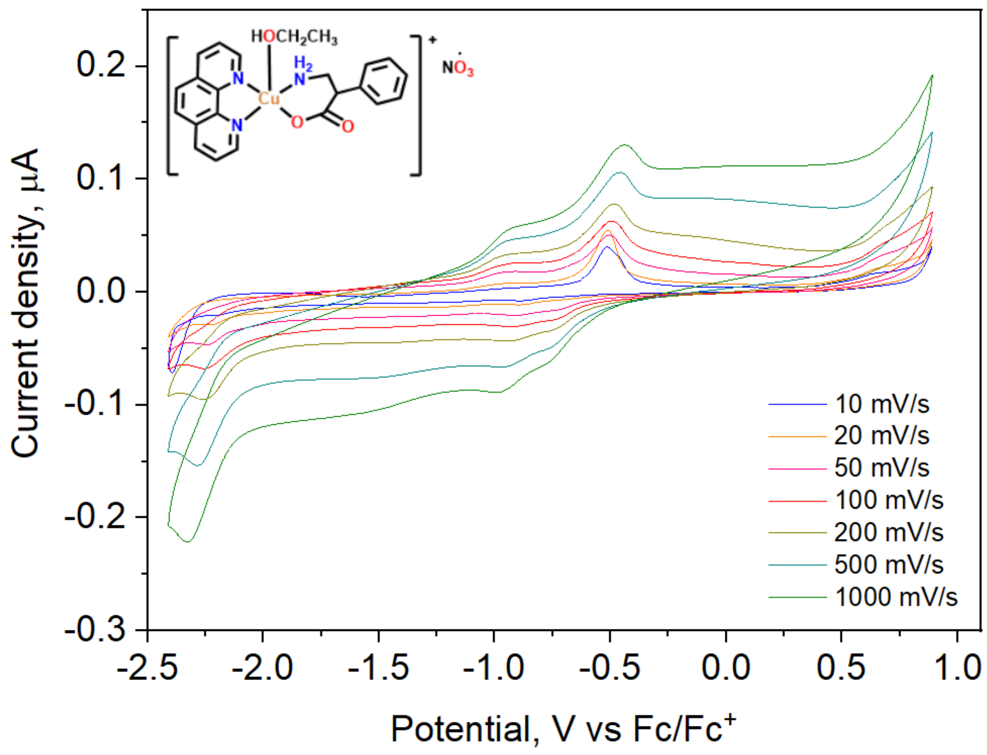

Figure S25. Half-wave potential for [Cu(EtOH)(phen)L2]NO_3 (**4**).

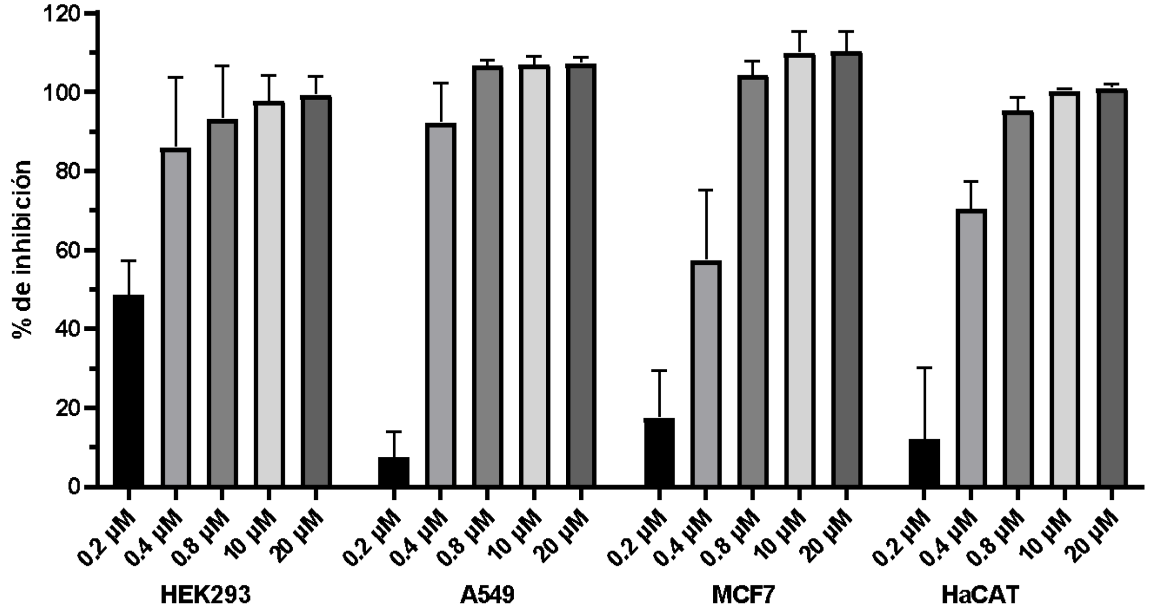

Figure S26. Half-maximal inhibitory concentration for [Cu(H_2O)(phen)L1]$NO_3 \cdot 2H_2O$ (**3**).

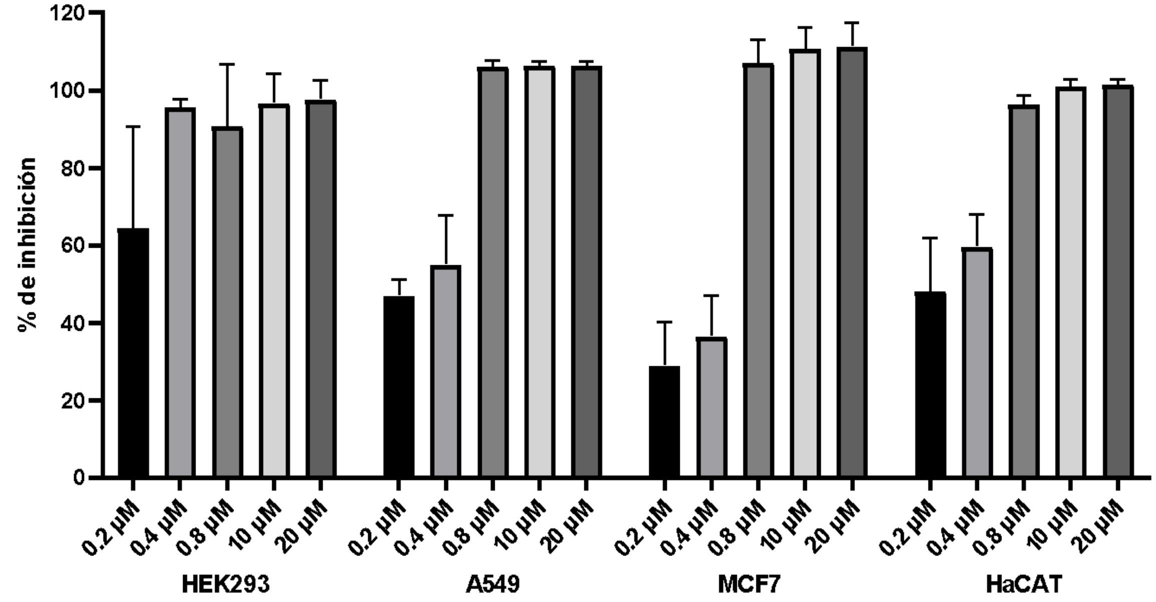

Figure S27. Half-maximal inhibitory concentration for [Cu(EtOH)(phen)L2]NO_3 (**4**).

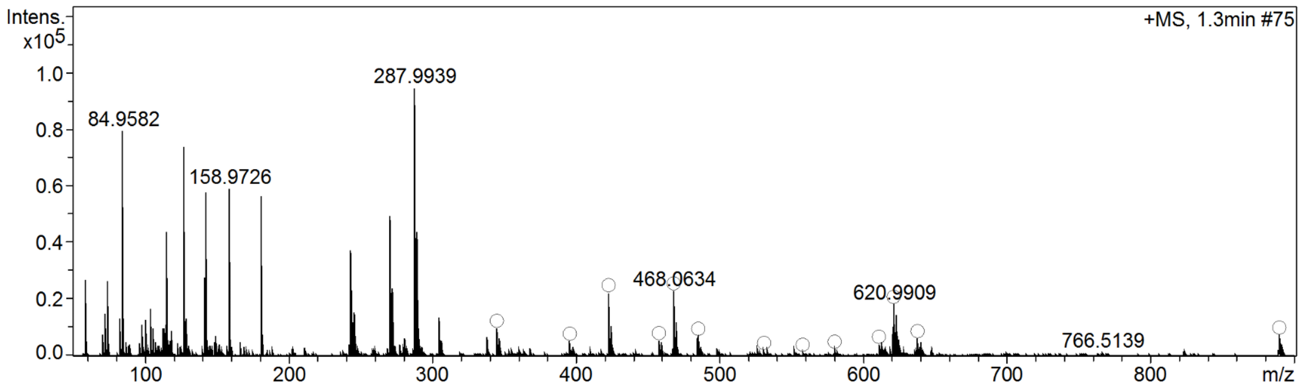

Meas. m/z	#	Ion Formula	Sum Formula	m/z	err [ppm]	|err| [mDa]	mSigma	# mSigma
345.0522	1	C16H16CuN3O2	C16H16CuN3O2	345.0533	3.1	1.1	142.8	1

Figure S28. HR MS(ESI$^+$) [Cu(H_2O)(phen)L1]NO_3·$2H_2O$ (**3**).

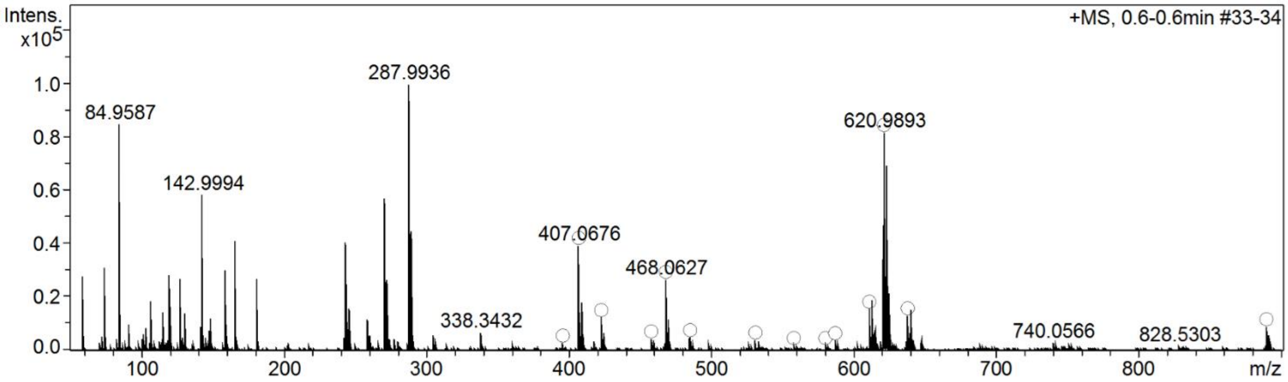

Meas. m/z	#	Ion Formula	Sum Formula	m/z	err [ppm]	|err| [mDa]	mSigma	# mSigma
407.0676	1	C21H18CuN3O2	C21H17CuN3O2	407.0690	3.3	1.4	61.2	3

Figure S29. HR MS(ESI$^+$) [Cu(EtOH)(phen)L2]NO_3 (**4**).

Table S1. Crystal data for [Cu(H_2O)(phen)L1]NO_3·2H_2O (**3**).

**Parameters**	Aqua(1,10-phenanthroline-κ^2*N,N'*)-(β^2-methylalaninate-κ^2*N,O*)copper(II) nitrate
**Empirical formula**	$C_{32}H_{42}N_8O_{15}Cu_2$
**Formula weight**	905.81
**Temperature (K)**	297.65
**Wavelength (Å)**	0.71073
**Crystal system**	Monoclinic
**Space group**	C2/c
**a (Å)**	19.8787(17)
**b (Å)**	7.1393(6)
**c (Å)**	26.896(2)
**α (°)**	90
**β(°)**	99.395(2)
**γ(°)**	90
**Volume (Å^3)**	3765.9(5)
**Z**	4
**D_{calc} (mg/m^3)**	1.594
**Absorption coefficient (μ/mm^{-1})**	1.211
**F(000)**	1872.0
**Crystal size (mm^3)**	0.46 × 0.34 × 0.29
**Theta range for data collection (°)**	6.072 to 61.228
**Index ranges**	$-28 \le h \le 28$<br>$-10 \le k \le 10$<br>$-38 \le l \le 38$
**Reflections collected**	60265

**Independent reflections**	5787 [R_{int} = 0.0595, R_{sigma} = 0.0311]
**Refinement method**	Full-matrix least-squares on F^2
**Data/restraints/parameters**	5787/2/283
**Goodness-of-fit on F2**	1.038
**Final R indices (I > 2sigma(I))**	R_1 = 0.0519, wR_2 = 0.1374
**Final R indices (all data)**	R_1 =0.0716, wR_2 = 0.1523
**Largest diff. peak and hole e Å^{-3}**	1.17/-0.92

Table S2. Bond Lengths for [Cu(H_2O)(phen)L1]NO_3·$2H_2O$ (**3**).

Atom	Atom	Length Å		Atom	Atom	Length Å
**Cu1**	N2	2.023(2)		C1	C2	1.397(4)
**Cu1**	O1	1.9180(19)		C2	C3	1.357(5)
**Cu1**	O3	2.295(3)		C3	C4	1.399(5)
**Cu1**	N3	1.976(2)		C4	C12	1.400(3)
**Cu1**	N1	2.026(2)		C4	C5	1.436(4)
**O4**	N4	1.163(4)		C12	C11	1.430(4)
**N2**	C11	1.354(3)		C11	C7	1.402(4)
**N2**	C10	1.325(4)		C7	C8	1.400(5)
**O6**	N4	1.183(5)		C7	C6	1.430(4)
**O1**	C13	1.275(3)		C8	C9	1.365(5)
**N4**	O5	1.174(5)		C9	C10	1.399(4)
**O2**	C13	1.237(3)		C6	C5	1.351(5)
**N3**	C15	1.473(4)		C15	C14	1.526(5)
**N1**	C1	1.325(4)		C14	C16	1.510(4)
**N1**	C12	1.354(3)		C14	C13	1.524(4)

Table S3. Bond Angles for [Cu(H_2O)(phen)L1]NO_3·$2H_2O$ (**3**).

Atom	Atom	Atom	Angle ˚		Atom	Atom	Atom	Angle ˚
**N2**	Cu1	O3	101.89(10)		C3	C4	C12	117.0(3)
**N2**	Cu1	N1	81.56(9)		C3	C4	C5	124.5(3)
**O1**	Cu1	N2	89.45(9)		C12	C4	C5	118.5(3)
**O1**	Cu1	O3	92.96(10)		N1	C12	C4	123.2(3)
**O1**	Cu1	N3	95.04(9)		N1	C12	C11	116.7(2)
**O1**	Cu1	N1	170.44(9)		C4	C12	C11	120.1(2)
**N3**	Cu1	N2	155.59(12)		N2	C11	C12	116.6(2)
**N3**	Cu1	O3	101.82(12)		N2	C11	C7	123.2(3)
**N3**	Cu1	N1	94.50(9)		C7	C11	C12	120.2(2)
**N1**	Cu1	O3	85.78(10)		C11	C7	C6	118.6(3)
**C11**	N2	Cu1	112.65(18)		C8	C7	C11	117.1(3)
**C10**	N2	Cu1	128.8(2)		C8	C7	C6	124.3(3)
**C10**	N2	C11	118.5(2)		C9	C8	C7	119.2(3)

**C13**	O1	Cu1	128.65(18)		C8	C9	C10	120.3(3)
**O4**	N4	O6	116.7(5)		N2	C10	C9	121.7(3)
**O4**	N4	O5	122.1(5)		C5	C6	C7	121.3(3)
**O5**	N4	O6	120.7(6)		C6	C5	C4	121.3(3)
**C15**	N3	Cu1	115.12(18)		N3	C15	C14	110.0(3)
**C1**	N1	Cu1	129.29(19)		C16	C14	C15	112.5(3)
**C1**	N1	C12	118.1(2)		C16	C14	C13	113.3(3)
**C12**	N1	Cu1	112.52(17)		C13	C14	C15	110.3(3)
**N1**	C1	C2	122.2(3)		O1	C13	C14	117.9(2)
**C3**	C2	C1	119.9(3)		O2	C13	O1	121.2(3)
**C2**	C3	C4	119.7(3)		O2	C13	C14	120.9(2)

Table S4. Torsion Angles for $[Cu(H_2O)(phen)L1]NO_3{\cdot}2H_2O$ (**3**).

A	B	C	D	Angle ˚		A	B	C	D	Angle ˚
**Cu1**	N2	C11	C12	-1.0(3)		C3	C4	C5	C6	-178.3(3)
**Cu1**	N2	C11	C7	-179.8(2)		C4	C12	C11	N2	-178.0(2)
**Cu1**	N2	C10	C9	-179.4(2)		C4	C12	C11	C7	0.9(4)
**Cu1**	O1	C13	O2	169.1(2)		C12	N1	C1	C2	-0.4(5)
**Cu1**	O1	C13	C14	-7.9(4)		C12	C4	C5	C6	1.4(5)
**Cu1**	N3	C15	C14	-53.2(3)		C12	C11	C7	C8	-179.5(3)
**Cu1**	N1	C1	C2	-177.7(3)		C12	C11	C7	C6	0.2(4)
**Cu1**	N1	C12	C4	178.6(2)		C11	N2	C10	C9	0.1(4)
**Cu1**	N1	C12	C11	0.0(3)		C11	C7	C8	C9	0.1(4)
**N2**	C11	C7	C8	-0.7(4)		C11	C7	C6	C5	-0.4(5)
**N2**	C11	C7	C6	179.0(3)		C7	C8	C9	C10	0.5(5)
**N3**	C15	C14	C16	-154.8(3)		C7	C6	C5	C4	-0.3(5)
**N3**	C15	C14	C13	77.6(4)		C8	C7	C6	C5	179.3(3)
**N1**	C1	C2	C3	-0.3(6)		C8	C9	C10	N2	-0.6(5)
**N1**	C12	C11	N2	0.7(4)		C10	N2	C11	C12	179.5(3)
**N1**	C12	C11	C7	179.5(2)		C10	N2	C11	C7	0.6(4)
**C1**	N1	C12	C4	0.8(4)		C6	C7	C8	C9	-179.6(3)
**C1**	N1	C12	C11	-177.8(3)		C5	C4	C12	N1	179.8(3)
**C1**	C2	C3	C4	0.6(6)		C5	C4	C12	C11	-1.7(4)
**C2**	C3	C4	C12	-0.2(5)		C15	C14	C13	O1	-44.7(4)
**C2**	C3	C4	C5	179.4(3)		C15	C14	C13	O2	138.3(3)
**C3**	C4	C12	N1	-0.5(4)		C16	C14	C13	O1	-171.9(4)
**C3**	C4	C12	C11	178.1(3)		C16	C14	C13	O2	11.1(5)

Table S5. Crystal data and structure refinement for [Cu(EtOH)(phen)L2]NO_3 (**4**).

Parameters	Ethanol(1,10-phenanthroline-κ^2*N,N'*)-(β^2-phenylalaninate-κ^2*N,O*)copper(II) nitrate
**Empirical formula**	$C_{23}H_{24}N_4O_6Cu$
**Formula weight**	516.016
**Temperature (K)**	100
**Wavelength (Å)**	0.71073
**Crystal system**	Monoclinic
**Space group**	P21/c
**a (Å)**	9.720(1)
**b (Å)**	18.6745(18)
**c (Å)**	11.7891(11)
**α (°)**	90
**β(°)**	97.045(3)
**γ(°)**	90
**Volume (Å^3)**	2123.8(4)
**Z**	4
**D_{calc} (mg/m^3)**	1.614
**Absorption coefficient (mm^{-1})**	1.080
**F(000)**	1070.2
**Crystal size (mm^3)**	0.42 × 0.4 × 0.19
**Theta range for data collection (°)**	4.36 to 55.96
**Index ranges**	$-12 \le h \le 12$<br>$-24 \le k \le 24$<br>$-15 \le l \le 15$
**Reflections collected**	80773
**Independent reflections**	5102 [Rint = 0.0491, Rsigma = 0.0196]
**Refinement method**	Full-matrix least-squares on F^2
**Data/restraints/parameters**	5102/1/315
**Goodness-of-fit on F2**	1.175
**Final R indices (I > 2sigma(I))**	R1 = 0.0511, wR2 = 0.1453
**Final R indices (all data)**	R1 = 0.0588, wR2 = 0.1503
**Largest diff. peak and hole e Å^{-3}**	1.10/−1.15

Table S6. Bond Lengths for [Cu(EtOH)(phen)L2]NO_3 (**4**).

**Atom**	**Atom**	**Length/Å**		**Atom**	**Atom**	**Length/Å**
**Cu1**	O2	1.956(2)		C4	C5	1.434(4)
**Cu1**	O3	2.223(2)		C4	C12	1.397(4)
**Cu1**	N1	2.063(3)		C5	C6	1.356(5)

**Cu1**	N2	2.003(2)		C6	C7	1.430(4)
**Cu1**	N3	1.966(3)		C7	C8	1.406(4)
**O1**	C21	1.243(3)		C7	C11	1.411(4)
**O2**	C21	1.278(3)		C8	C12	1.436(4)
**O3**	C22	1.436(6)		C9	C10	1.401(4)
**O4**	N4	1.235(4)		C10	C11	1.369(5)
**O5**	N4	1.251(4)		C13	C14	1.520(4)
**O6**	N4	1.251(3)		C14	C15	1.520(4)
**N1**	C1	1.331(4)		C14	C21	1.544(4)
**N1**	C12	1.360(4)		C15	C16	1.386(4)
**N2**	C8	1.351(4)		C15	C20	1.386(4)
**N2**	C9	1.325(4)		C16	C17	1.387(5)
**N3**	C13	1.470(4)		C17	C18	1.368(5)
**C1**	C2	1.399(4)		C18	C19	1.372(5)
**C2**	C3	1.375(5)		C19	C20	1.389(5)
**C3**	C4	1.412(4)		C22	C23	1.569(9)

Table S7. Bond Angles for [Cu(EtOH)(phen)L2]NO_3 (**4**).

**Atom**	**Atom**	**Atom**	**Angle °**		**Atom**	**Atom**	**Atom**	**Angle °**
**O3**	Cu1	O2	95.45(9)		C7	C6	C5	121.2(3)
**N1**	Cu1	O2	166.77(9)		C8	C7	C6	118.7(3)
**N1**	Cu1	O3	94.58(9)		C11	C7	C6	124.3(3)
**N2**	Cu1	O2	89.61(10)		C11	C7	C8	117.0(3)
**N2**	Cu1	O3	92.89(10)		C7	C8	N2	123.3(3)
**N2**	Cu1	N1	81.28(10)		C12	C8	N2	116.6(2)
**N3**	Cu1	O2	93.05(10)		C12	C8	C7	120.1(3)
**N3**	Cu1	O3	93.27(11)		C10	C9	N2	122.1(3)
**N3**	Cu1	N1	94.97(10)		C11	C10	C9	120.0(3)
**N3**	Cu1	N2	173.02(12)		C10	C11	C7	119.1(3)
**C21**	O2	Cu1	125.96(19)		C4	C12	N1	123.8(3)
**C22**	O3	Cu1	120.2(3)		C8	C12	N1	116.5(3)
**C1**	N1	Cu1	130.4(2)		C8	C12	C4	119.7(3)
**C12**	N1	Cu1	111.69(18)		C14	C13	N3	112.5(3)
**C12**	N1	C1	117.8(3)		C15	C14	C13	110.6(2)
**C8**	N2	Cu1	113.90(19)		C21	C14	C13	113.4(2)
**C9**	N2	Cu1	127.5(2)		C21	C14	C15	111.3(2)
**C9**	N2	C8	118.5(3)		C16	C15	C14	120.2(3)
**C13**	N3	Cu1	114.0(2)		C20	C15	C14	121.7(3)
**O5**	N4	O4	121.0(3)		C20	C15	C16	118.0(3)
**O6**	N4	O4	120.5(3)		C17	C16	C15	120.9(3)
**O6**	N4	O5	118.5(3)		C18	C17	C16	120.5(3)
**C2**	C1	N1	122.5(3)		C19	C18	C17	119.4(3)
**C3**	C2	C1	119.7(3)		C20	C19	C18	120.5(3)
**C4**	C3	C2	119.3(3)		C19	C20	C15	120.6(3)
**C5**	C4	C3	123.8(3)		O2	C21	O1	123.1(3)

**C12**	C4	C3	116.9(3)		C14	C21	O1	117.5(3)
**C12**	C4	C5	119.2(3)		C14	C21	O2	119.4(2)
**C6**	C5	C4	120.9(3)		C23	C22	O3	140.3(5)

Table S8. Torsion Angles for [Cu(EtOH)(phen)L2]NO_3 (**4**).

**A**	**B**	**C**	**D**	**Angle ˚**		**A**	**B**	**C**	**D**	**Angle ˚**
**Cu1**	O2	C21	O1	139.8(2)		N3	C13	C14	C15	-171.3(3)
**Cu1**	O2	C21	C14	-39.0(2)		N3	C13	C14	C21	62.7(3)
**Cu1**	O3	C22	C23	169.7(4)		C1	C2	C3	C4	0.4(4)
**Cu1**	N1	C1	C2	177.7(3)		C2	C3	C4	C5	179.5(3)
**Cu1**	N1	C12	C4	-178.12(18)		C2	C3	C4	C12	-0.4(3)
**Cu1**	N1	C12	C8	1.1(2)		C3	C4	C5	C6	179.7(3)
**Cu1**	N2	C8	C7	178.81(18)		C3	C4	C12	C8	-179.0(3)
**Cu1**	N2	C8	C12	-0.4(2)		C4	C5	C6	C7	-0.8(4)
**Cu1**	N2	C9	C10	-178.2(3)		C4	C12	C8	C7	-0.5(3)
**Cu1**	N3	C13	C14	-64.3(2)		C5	C6	C7	C8	1.5(4)
**O1**	C21	C14	C13	172.6(3)		C5	C6	C7	C11	-177.6(3)
**O1**	C21	C14	C15	47.1(3)		C6	C7	C8	C12	-0.8(3)
**O2**	C21	C14	C13	-8.6(3)		C6	C7	C11	C10	179.1(3)
**O2**	C21	C14	C15	-134.1(3)		C7	C11	C10	C9	0.2(4)
**N1**	C1	C2	C3	-0.2(4)		C13	C14	C15	C16	134.9(3)
**N1**	C12	C4	C3	0.2(3)		C13	C14	C15	C20	-43.5(3)
**N1**	C12	C4	C5	-179.7(3)		C14	C15	C16	C17	-176.1(3)
**N1**	C12	C8	N2	-0.5(3)		C14	C15	C20	C19	176.0(3)
**N1**	C12	C8	C7	-179.7(2)		C15	C16	C17	C18	-0.4(3)
**N2**	C8	C7	C6	-179.9(3)		C15	C20	C19	C18	0.7(4)
**N2**	C8	C7	C11	-0.8(3)		C16	C17	C18	C19	-1.4(4)
**N2**	C8	C12	C4	178.7(3)		C17	C18	C19	C20	1.3(4)
**N2**	C9	C10	C11	0.4(4)						

CheckCIF for [Cu(H_2O)(phen)L1]NO_3·2H_2O (**3**).

1 checkCIF/PLATON (basic structural check)

Structure factors have been supplied for datablock(s) ejjaime026_0m

THIS REPORT IS FOR GUIDANCE ONLY. IF USED AS PART OF A REVIEW PROCEDURE FOR PUBLICATION, IT SHOULD NOT REPLACE THE EXPERTISE OF AN EXPERIENCED CRYSTALLOGRAPHIC REFEREE.

No syntax errors found. CIF dictionary
Please wait while processing .... Interpreting this report
Structure factor report

Datablock: ejjaime026_0m

Bond precision:	C-C = 0.0046 A		Wavelength=0.71073
Cell:	a=19.8787(17)	b=7.1393(6)	c=26.896(2)
	alpha=90	beta=99.395(2)	gamma=90
Temperature:	298 K		

	Calculated	Reported
Volume	3765.9(5)	3765.9(5)
Space group	C 2/c	C 1 2/c 1
Hall group	-C 2yc	-C 2yc
Moiety formula	2(C16 H18 Cu N3 O3), 2(N O3), 3(H2 O)	2(C16 H18 Cu N3 O3), 2(N O3), 3(H2 O)
Sum formula	C32 H42 Cu2 N8 O15	C32 H42 Cu2 N8 O15
Mr	905.84	905.81
Dx,g cm-3	1.598	1.598
Z	4	4
Mu (mm-1)	1.211	1.211
F000	1872.0	1872.0
F000'	1875.41	

h,k,lmax	28,10,38	28,10,38
Nref	5794	5787
Tmin,Tmax	0.616,0.704	
Tmin'	0.567	

Correction method= Not given

Data completeness= 0.999   Theta(max)= 30.614

R(reflections)= 0.0519(4372)   wR2(reflections)= 0.1523(5787)

S = 1.038   Npar= 287

The following ALERTS were generated. Each ALERT has the format
test-name_ALERT_alert-type_alert-level.
Click on the hyperlinks for more details of the test.

Alert level C

PLAT052_ALERT_1_C Info on Absorption Correction Method Not Given Please Do !
PLAT057_ALERT_3_C Correction for Absorption Required RT(exp) ... 1.14 Do !
PLAT244_ALERT_4_C Low 'Solvent' Ueq as Compared to Neighbors of N4 Check
PLAT260_ALERT_2_C Large Average Ueq of Residue Including O4 0.140 Check
PLAT911_ALERT_3_C Missing FCF Refl Between Thmin & STh/L= 0.600 2 Report
0 2 0, 2 2 0,

Alert level G

PLAT002_ALERT_2_G Number of Distance or Angle Restraints on AtSite 3 Note
PLAT083_ALERT_2_G SHELXL Second Parameter in WGHT Unusually Large 8.68 Why ?
PLAT172_ALERT_4_G The CIF-Embedded .res File Contains DFIX Records 1 Report
PLAT790_ALERT_4_G Centre of Gravity not Within Unit Cell: Resd. # 3 Note
H2 O
PLAT794_ALERT_5_G Tentative Bond Valency for Cu1 (II) . 2.21 Info
PLAT860_ALERT_3_G Number of Least-Squares Restraints .... 2 Note

```
PLAT883_ALERT_1_G No Info/Value for _atom_sites_solution_primary .
Please Do !
PLAT910_ALERT_3_G Missing # of FCF Reflection(s) Below Theta(Min).
4 Note
                  2   0   0,   -2   0   2,    0   0   2,    2   0   2,
PLAT912_ALERT_4_G Missing # of FCF Reflections Above STh/L=  0.600
2 Note
PLAT913_ALERT_3_G Missing # of Very Strong Reflections in FCF ....
2 Note
                  0   2   0,    2   2   0,
PLAT969_ALERT_5_G The 'Henn et al.' R-Factor-gap value ...........
4.26 Note
                Predicted wR2: Based on SigI**2  3.58 or SHELX Weight 15.05
PLAT978_ALERT_2_G Number C-C Bonds with Positive Residual Density.
4 Info
```

```
   0 ALERT level A = Most likely a serious problem - resolve or explain
   0 ALERT level B = A potentially serious problem, consider carefully
   5 ALERT level C = Check. Ensure it is not caused by an omission or
oversight
  12 ALERT level G = General information/check it is not something
unexpected

   2 ALERT type 1 CIF construction/syntax error, inconsistent or missing
data
   4 ALERT type 2 Indicator that the structure model may be wrong or
deficient
   5 ALERT type 3 Indicator that the structure quality may be low
   4 ALERT type 4 Improvement, methodology, query or suggestion
   2 ALERT type 5 Informative message, check
```

It is advisable to attempt to resolve as many as possible of the alerts in all categories. Often the minor alerts point to easily fixed oversights, errors and omissions in your CIF or refinement strategy, so attention to these fine details can be worthwhile. In order to resolve some of the more serious problems it may be necessary to carry out additional measurements or structure refinements. However, the purpose of your study may justify the reported deviations and the more serious of these should normally be commented upon in the discussion or experimental section of a paper or in the "special_details" fields of the CIF. checkCIF was carefully designed to identify outliers and unusual parameters, but every test has its limitations and alerts that are not important in a particular case may appear. Conversely, the absence of alerts does not guarantee there are no aspects of the results needing attention. It is up to the individual to critically assess their own results and, if necessary, seek expert advice.

Publication of your CIF in IUCr journals

A basic structural check has been run on your CIF. These basic checks will be run on all CIFs submitted for publication in IUCr journals (*Acta Crystallographica*, *Journal of Applied Crystallography*, *Journal of Synchrotron Radiation*); however, if you intend to submit to *Acta Crystallographica Section C* or *E* or *IUCrData*, you should make sure that full publication checks are run on the final version of your CIF prior to submission.

Publication of your CIF in other journals

Please refer to the *Notes for Authors* of the relevant journal for any special instructions relating to CIF submission.

PLATON version of 06/01/2024; check.def file version of 05/01/2024

Datablock ejjaime026_0m - ellipsoid plot

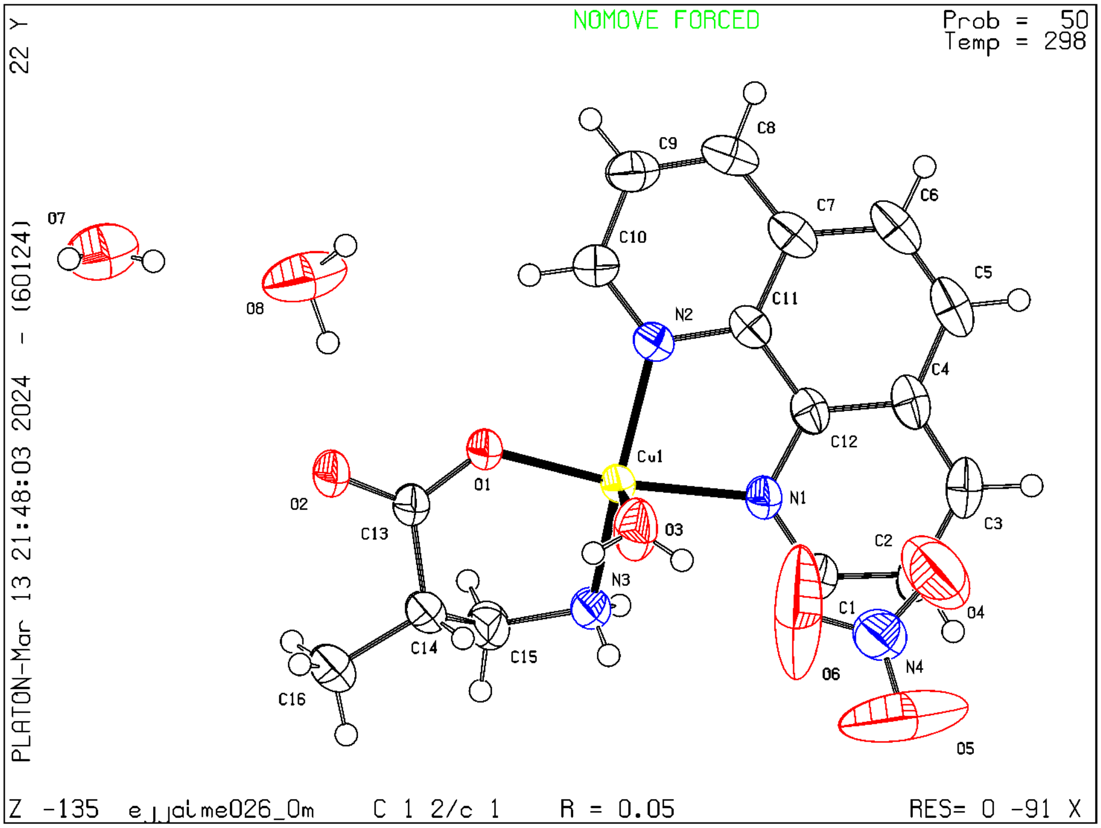

CheckCIF for [Cu(EtOH)(phen)L2]NO_3 (**4**).

2 checkCIF/PLATON (basic structural check)

Structure factors have been supplied for datablock(s) ejgonzalo05_0m

THIS REPORT IS FOR GUIDANCE ONLY. IF USED AS PART OF A REVIEW PROCEDURE FOR PUBLICATION, IT SHOULD NOT REPLACE THE EXPERTISE OF AN EXPERIENCED CRYSTALLOGRAPHIC REFEREE.

No syntax errors found. CIF dictionary
Please wait while processing .... Interpreting this report
Structure factor report

Datablock: ejgonzalo05_0m

Bond precision:	C-C = 0.0042 A		Wavelength=0.71073
Cell:	a=9.720(1)	b=18.6745(18)	c=11.7891(11)
	alpha=90	beta=97.045(3)	gamma=90
Temperature:	100 K		

	Calculated	Reported
Volume	2123.8(4)	2123.8(4)
Space group	P 21/c	P 1 21/c 1
Hall group	-P 2ybc	-P 2ybc
Moiety formula	C23 H24 Cu N4 O6	C23 H24 Cu N3 O3, N O3
Sum formula	C23 H24 Cu N4 O6	C23 H24 Cu N4 O6
Mr	516.01	516.00
Dx,g cm-3	1.614	1.614
Z	4	4
Mu (mm-1)	1.080	1.080
F000	1068.0	1068.0
F000'	1069.71	

h,k,lmax	12,24,15	12,24,15
Nref	5118	5080
Tmin,Tmax	0.789,0.814	0.608,0.746
Tmin'	0.789	

Correction method= # Reported T Limits: Tmin=0.608 Tmax=0.746 AbsCorr = NONE

Data completeness= 0.993   Theta(max)= 27.984

R(reflections)= 0.0463(4425)   wR2(reflections)= 0.1248(5080)

S = 1.098   Npar= 315

The following ALERTS were generated. Each ALERT has the format
test-name_ALERT_alert-type_alert-level.
Click on the hyperlinks for more details of the test.

Alert level B

PLAT230_ALERT_2_B Hirshfeld Test Diff for O3 --C22 . 15.0 s.u.
PLAT780_ALERT_1_B Coordinates do not Form a Properly Connected Set Please Do !

Alert level C

PLAT042_ALERT_1_C Calc. and Reported MoietyFormula Strings Differ Please Check
Calc: C23 H24 Cu N4 O6
Rep.: C23 H24 Cu N3 O3, N O3
PLAT220_ALERT_2_C NonSolvent Resd 1 C Ueq(max)/Ueq(min) Range 3.2 Ratio
PLAT222_ALERT_3_C NonSolvent Resd 1 H Uiso(max)/Uiso(min) Range 4.2 Ratio
PLAT241_ALERT_2_C High 'MainMol' Ueq as Compared to Neighbors of C22 Check
PLAT242_ALERT_2_C Low 'MainMol' Ueq as Compared to Neighbors of O3 Check
PLAT242_ALERT_2_C Low 'MainMol' Ueq as Compared to Neighbors of N4 Check
PLAT413_ALERT_2_C Short Inter XH3 .. XHn H9 ..H23B . 2.11 Ang.
x,1/2-y,-1/2+z = 4_565 Check
PLAT906_ALERT_3_C Large K Value in the Analysis of Variance ...... 2.659 Check

PLAT911_ALERT_3_C Missing FCF Refl Between Thmin & STh/L= 0.600
24 Report
2 1 0, 1 2 0, -2 0 2, 0 0 2, 3 0 2, 0
1 2,
-2 2 2, 1 2 2, 4 2 2, -5 3 2, 0 3 2, 1
3 2,
-2 1 3, -5 1 4, 2 2 4, -8 7 5, -6 0 6, -5
1 6,
-3 1 6, -1 3 6, 3 9 6, 0 0 8, -5 1 8, -3
6 9,
PLAT913_ALERT_3_C Missing # of Very Strong Reflections in FCF ....
4 Note
-2 0 2, 0 0 2, 1 3 2, -2 1 3,

Alert level G

PLAT002_ALERT_2_G Number of Distance or Angle Restraints on AtSite
5 Note
PLAT004_ALERT_5_G Polymeric Structure Found with Maximum Dimension
1 Info
PLAT171_ALERT_4_G The CIF-Embedded .res File Contains EADP Records
1 Report
PLAT172_ALERT_4_G The CIF-Embedded .res File Contains DFIX Records
2 Report
PLAT187_ALERT_4_G The CIF-Embedded .res File Contains RIGU Records
1 Report
PLAT190_ALERT_3_G A Non-default RIGU Restraint Value for First Par
0.0000 Report
PLAT190_ALERT_3_G A Non-default RIGU Restraint Value for SecondPar
0.0000 Report
PLAT343_ALERT_2_G Unusual sp3 Angle Range in Main Residue for
C22 Check
PLAT432_ALERT_2_G Short Inter X...Y Contact O4 ..C23 .
2.94 Ang.
x,1/2-y,-1/2+z = 4_565
Check
PLAT794_ALERT_5_G Tentative Bond Valency for Cu1 (II) .
2.20 Info
PLAT860_ALERT_3_G Number of Least-Squares Restraints ....
6 Note
PLAT910_ALERT_3_G Missing # of FCF Reflection(s) Below Theta(Min).
4 Note
1 0 0, 1 1 0, 0 2 0, 0 1 1,
PLAT912_ALERT_4_G Missing # of FCF Reflections Above STh/L= 0.600
10 Note
PLAT933_ALERT_2_G Number of HKL-OMIT Records in Embedded .res File
22 Note
-8 7 5, -6 0 6, -5 1 4, -5 1 6, -5 1 8, -5
3 2,
-3 1 6, -3 6 9, -2 2 2, -1 3 6, 0 0 8, 0
1 2,
0 2 0, 0 3 2, 1 1 0, 1 2 0, 1 2 2, 2
1 0,
2 2 4, 3 0 2, 3 9 6, 4 2 2,
PLAT969_ALERT_5_G The 'Henn et al.' R-Factor-gap value ....
5.84 Note
Predicted wR2: Based on SigI**2 2.14 or SHELX Weight 11.74

```
PLAT978_ALERT_2_G Number C-C Bonds with Positive Residual Density.
9 Info
```

```
   0 ALERT level A = Most likely a serious problem - resolve or explain
   2 ALERT level B = A potentially serious problem, consider carefully
  10 ALERT level C = Check. Ensure it is not caused by an omission or
oversight
  16 ALERT level G = General information/check it is not something
unexpected

   2 ALERT type 1 CIF construction/syntax error, inconsistent or missing
data
  11 ALERT type 2 Indicator that the structure model may be wrong or
deficient
   8 ALERT type 3 Indicator that the structure quality may be low
   4 ALERT type 4 Improvement, methodology, query or suggestion
   3 ALERT type 5 Informative message, check
```

Validation response form

Please find below a validation response form (VRF) that can be filled in and pasted into your CIF.

```
# start Validation Reply Form
_vrf_PLAT230_ejgonzalo05_0m
;
PROBLEM: Hirshfeld Test Diff for    O3        --C22       .       15.0 s.u.
RESPONSE: Some atoms are not ideally shaped but this not indicate an
incorrect atom-type assignment.
;
_vrf_PLAT780_ejgonzalo05_0m
;
PROBLEM: Coordinates do not Form a Properly Connected Set     Please Do !
RESPONSE: This alert is related to disorder of solvent and anions. These
kinds of alerts are acceptable from crystallographic point of view.
;
# end Validation Reply Form
```

It is advisable to attempt to resolve as many as possible of the alerts in all categories. Often the minor alerts point to easily fixed oversights, errors and omissions in your CIF or refinement strategy, so attention to these fine details can be worthwhile. In order to resolve some of the more serious problems it may be necessary to carry out additional measurements or structure refinements. However, the purpose of your study may justify the reported deviations and the more serious of these should normally be commented upon in the discussion or experimental section of a paper or in the "special_details" fields of the CIF. checkCIF was carefully designed to identify outliers and unusual parameters, but every test has its limitations and alerts that are not important in a particular case may appear. Conversely, the absence of alerts does not guarantee there are no aspects of the results needing attention. It is up to the individual to critically assess their own results and, if necessary, seek expert advice.

Publication of your CIF in IUCr journals

A basic structural check has been run on your CIF. These basic checks will be run on all CIFs submitted for publication in IUCr journals (*Acta Crystallographica*, *Journal of Applied Crystallography*, *Journal of Synchrotron Radiation*); however, if you intend to submit to *Acta Crystallographica Section C* or *E* or *IUCrData*, you should make sure that full publication checks are run on the final version of your CIF prior to submission.

Publication of your CIF in other journals

Please refer to the *Notes for Authors* of the relevant journal for any special instructions relating to CIF submission.

PLATON version of 06/01/2024; check.def file version of 05/01/2024

Datablock ejgonzalo05_0m - ellipsoid plot

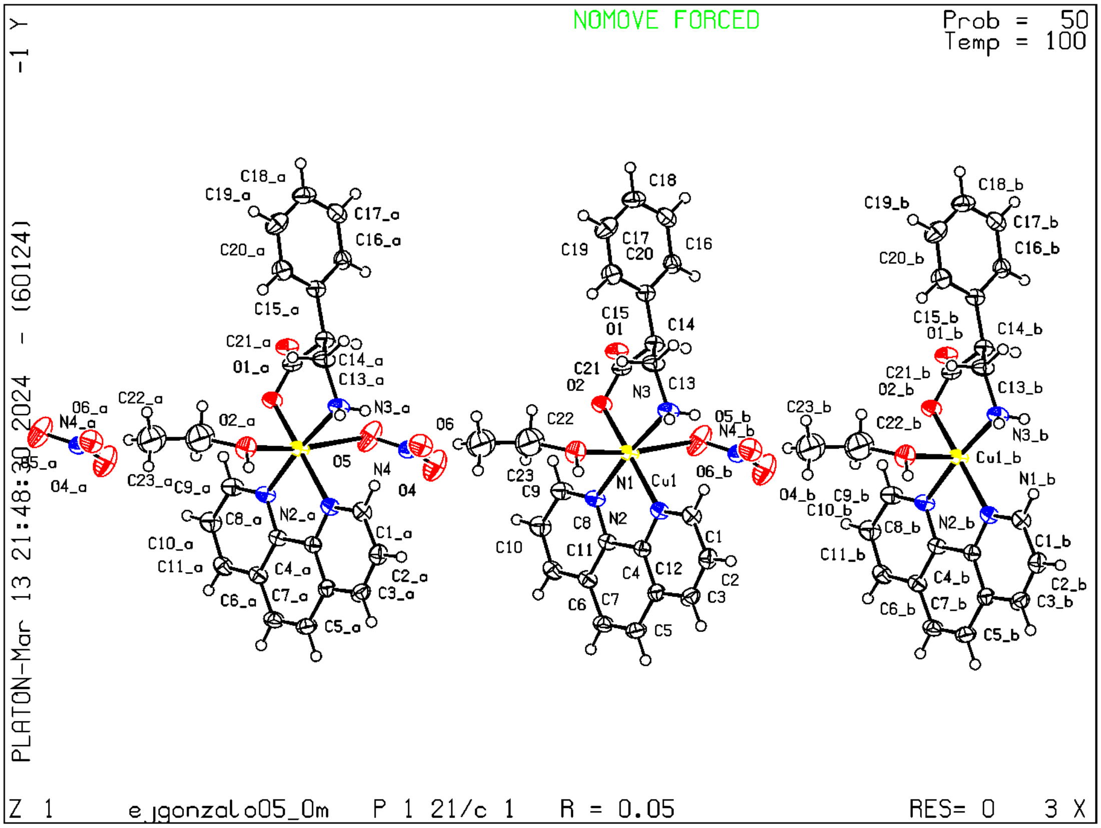

Supplement: Supplementary file 1 [file molecules-30-00634-s001.zip › molecules-3429203-supplementary.pdf]
